# Hyperinsulinemia and insulin resistance in the obese may develop as part of a homeostatic response to elevated free fatty acids: A mechanistic case-control and a population-based cohort study

**DOI:** 10.1016/j.ebiom.2021.103264

**Published:** 2021-03-09

**Authors:** Emanuel Fryk, Josefin Olausson, Karin Mossberg, Lena Strindberg, Martin Schmelz, Helén Brogren, Li-Ming Gan, Silvano Piazza, Alessandro Provenzani, Barbara Becattini, Lars Lind, Giovanni Solinas, Per-Anders Jansson

**Affiliations:** a The Wallenberg Laboratory and Sahlgrenska Center for Cardiovascular and Metabolic Research, Department of Molecular and Clinical Medicine, Institute of Medicine, University of Gothenburg, Gothenburg, Sweden; b Department of Public Health and Community Medicine, Institute of Medicine, University of Gothenburg, Gothenburg, Sweden; c Department of Anesthesiology and Intensive Care Medicine Mannheim, University of Heidelberg, Heidelberg Germany; d Department of Cardiology Sahlgrenska Center for Cardiovascular and Metabolic Research, Department of Molecular and Clinical Medicine, Institute of Medicine, University of Gothenburg, Gothenburg, Sweden; e Research and Early Development, Cardiovascular, Renal and Metabolism, BioPharmaceuticals R&D, AstraZeneca, Gothenburg, Sweden; f Centre for Integrative Biology, CIBIO, University of Trento, Trento Italy; g Computational Biology, International Centre for Genetic Engineering and Biotechnology, ICGEB, 34149 Trieste, Italy; h Dep of Medical Sciences, Uppsala University, Uppsala, Sweden

**Keywords:** Adaptive response, Adipose tissue, Free fatty acids, Insulin resistance, Lipolysis, Microdialysis, Obesity, RNA sequencing, Type 2 diabetes

## Abstract

**Background:**

It is commonly accepted that in obesity free fatty acids (FFA) cause insulin resistance and hyperglycemia, which drives hyperinsulinemia. However, hyperinsulinemia is observed in subjects with normoglycaemia and thus the paradigm above should be reevaluated.

**Methods:**

We describe two studies: MD-Lipolysis, a case control study investigating the mechanisms of obesity-driven insulin resistance by a systemic metabolic analysis, measurements of adipose tissue lipolysis by microdialysis, and adipose tissue genomics; and POEM, a cohort study used for validating differences in circulating metabolites in relation to adiposity and insulin resistance observed in the MD-Lipolysis study.

**Findings:**

In insulin-resistant obese with normal glycaemia from the MD-Lipolysis study, hyperinsulinemia was associated with elevated FFA. Lipolysis, assessed by glycerol release per adipose tissue mass or adipocyte surface, was similar between obese and lean individuals. Adipose tissue from obese subjects showed reduced expression of genes mediating catecholamine-driven lipolysis, lipid storage, and increased expression of genes driving hyperplastic growth. In the POEM study, FFA levels were specifically elevated in obese-overweight subjects with normal fasting glucose and high fasting levels of insulin and C-peptide.

**Interpretation:**

In obese subjects with normal glycaemia elevated circulating levels of FFA at fasting are the major metabolic derangement candidate driving fasting hyperinsulinemia. Elevated FFA in obese with normal glycaemia were better explained by increased fat mass rather than by adipose tissue insulin resistance. These results support the idea that hyperinsulinemia and insulin resistance may develop as part of a homeostatic adaptive response to increased adiposity and FFA.

**Funding:**

Swedish-Research-Council (2016-02660); Diabetesfonden (DIA2017-250; DIA2018-384; DIA2020-564); Novo-Nordisk-Foundation (NNF17OC0027458; NNF19OC0057174); Cancerfonden (CAN2017/472; 200840PjF); Swedish-ALF-agreement (2018-74560).

**Research in context**

**Evidence before this study**

It is commonly accepted that elevated free fatty acid (FFA) levels in obesity cause insulin resistance and that, as a consequence, increased blood glucose is driving hyperinsulinemia. However, pronounced hyperinsulinemia is observed in obese with normal glucose levels, indicating that high blood glucose is not the driver of hyperinsulinemia in these subjects. Furthermore, FFA are not generally elevated in obese subjects. Overall, the mechanisms initiating hyperinsulinemia and insulin resistance in obesity and the role of high FFA levels in this process remains largely unresolved.

**Added value of this study**

Our data from the MD-Lipolysis case control study and from the population-based POEM study indicate that elevated FFA are likely to be the metabolic derangement driving hyperinsulinemia in subjects with increased adiposity but normal glycaemic control.

Using the microdialysis technique we have found that, in hyperinsulinemic obese with normal plasma glucose, elevated FFA levels can mostly be explained by their increased fat mass rather than by an overt and uncompensated adipose tissue insulin resistance. Adipose tissue analysis of gene expression showed that obese individuals display a reduced expression of key genes mediating catecholamine-driven lipolysis, de-novo lipogenesis and lipid storage, but increased expression of genes implicated in adipose tissue hyperplasia.

**Implications of all of the available evidence**

We conclude that elevated fasting blood levels of FFA, rather than high glucose, is the major metabolic derangement initiating fasting hyperinsulinemia in obese subjects with normal glycaemic control. In these subjects, hyperinsulinemia and insulin resistance, together with changes in adipose tissue blood flow and gene expression, may develop as part of an adaptive response to increased adiposity and FFA levels.

Alt-text: Unlabelled box

## 1 Introduction

Excessive adiposity leads to hyperinsulinemia and insulin resistance, a major risk factor for diabetes mellitus and a cluster of related diseases collectively known as the metabolic syndrome [1]. It is commonly accepted that hyperinsulinemia is consequent to resistance to insulin action in glucose metabolism, leading to increased glycaemia, which in turn stimulates the pancreatic β-cell to release insulin to avoid a more severe hyperglycemia [2,3]. However, a major limitation of this model is that a marked hyperinsulinemia can be observed in subjects with normal glycemic control, suggesting that high blood glucose may not be the driver of hyperinsulinemia in these subjects [2]. Along this line of reasoning, it has been proposed that hyperinsulinemia itself may be a cause of the resistance to insulin action in glucose metabolism instead of being a consequence of it, as elevated basal levels of insulin are expected to desensitize insulin target cells to insulin stimulation [2,[4], [5], [6], [7]]. However, the metabolic changes driving insulin secretion in the pancreatic β-cell, which causes hyperinsulinemia in this latter model, remains to be identified.

Although, insulin secretion is typically regarded as a process chiefly controlled by blood glucose, it is well established that free fatty acids (FFA) can also stimulate the pancreatic β-cell to secrete insulin [8], [9], [10]. It is thus possible that elevated circulating levels of FFA from adipose tissue lipolysis, instead of hyperglycemia, constitute the metabolic derangement initiating fasting hyperinsulinemia in obesity. However, this hypothesis remains to be validated.

Cross-sectional and longitudinal studies indicate that fasting levels of circulating FFA are positively correlated with loss of glucose homeostasis and predict the incidence of type 2 diabetes [11], [12], [13], [14], [15], [16]. However, FFA are typically regarded as the cause of insulin resistance rather than a direct driver of insulin secretion by the β-cell. Indeed, with the exception of insulin-induced insulin resistance, virtually every molecular mechanism proposed to explain insulin resistance in obesity since the Randle glucose-FFA cycle, indicate elevated circulating FFA levels as a major driver of insulin resistance [2,3,[17], [18], [19], [20], [21], [22], [23], [24], [25]]. However, the quantitative contribution and the specific relevance of each of these mechanisms to the development of insulin resistance in obese humans remains unresolved. Moreover, the general role of FFA as pathogenic drivers of insulin resistance has been questioned: data on 5790 individuals from the Paris Prospective Study and on 1591 individuals from the Oxford Biobank indicate that fasting levels of circulating FFA are not generally elevated in subjects with larger fat mass [26]; and it was reported that FFA release per 100 g of adipose tissue are downregulated in subjects with larger fat mass and negatively correlated with fasting circulating insulin levels [26]. Collectively, these data suggest that FFA levels are not elevated in the general obese population, and that their adipose tissue is insulin sensitive.

Interestingly, recent studies show that, obese subjects with elevated fasting insulin, but normal fasting glycaemia display elevated FFA compared to lean control subjects [11,12,27]. These data are consistent with the idea that elevated FFA may directly drive fasting hyperinsulinemia, leading to insulin resistance. However, the interpretation of these studies is complicated by the limited number of participants [27]; or by an unbalanced female to male ratio with females being overrepresented in the obese groups compared to the lean groups [11,12]. Indeed, it is well established that females typically display higher levels of FFA than males [26].

Here we describe two studies: a case control study (MD-Lipolysis) investigating the mechanisms of obesity-driven insulin resistance by combining measurements of circulating levels of metabolites and insulin, adipose tissue lipolysis rates by microdialysis, with adipose tissue genomics; and a cohort study (POEM) to assess the validity of the observed circulating metabolites relation to adiposity and insulin resistance initially found in MD-Lipolysis study.

Collectively, our results are consistent with the idea that insulin resistance may develop in the context of an adaptive homeostatic response restraining the increase of circulating levels of FFA driven by larger adipose mass of obese subjects.

## 2 Methods

### 2.1 MD-Lipolysis study

#### 2.1.1 Study participants MD-Lipolysis study

**Fig. 1:**
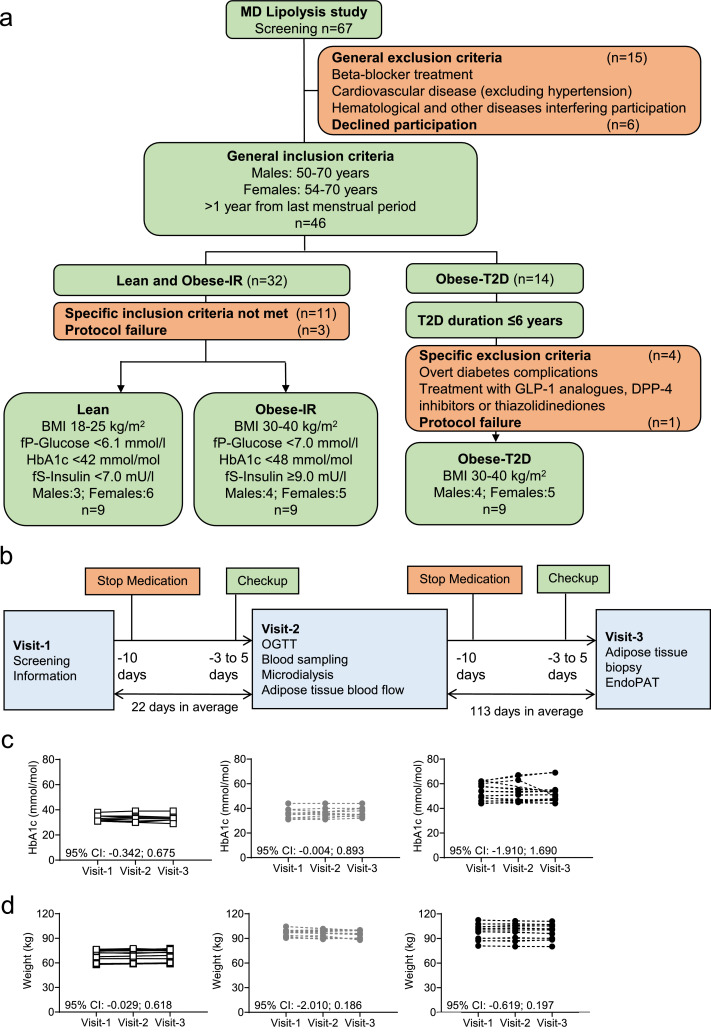
MD-Lipolysis study design and characteristics of subjects. (a) Illustration of the volunteer selection procedure for the MD-Lipolysis study by specific inclusion (green) and exclusion (red) criteria, which defined the experimental groups: Lean; Obese-IR; and Obese-T2D. (b) The experimental plan was implemented in three visits: screening and collection of information of participants was performed at visit-1; blood and abdominal subcutaneous adipose tissue interstitial dialysate fluids were collected after an overnight fasting and during an oral glucose tolerance test (OGTT), and abdominal subcutaneous adipose tissue blood flow (ATBF) measurements (by ^133^Xe-clearance technique) were performed at visit-2; EndoPAT (peripheral arterial tone) measurements of endothelial function and subcutaneous adipose tissue biopsies collection were performed at visit-3. (c) HbA1c values of participants, divided by experimental groups, at each study visit. (d) Weight of participants, divided by experimental groups, at each study visit. Data are presented as individual data points for each participant. n=9 for c and d, except for Visit-3 where one Obese-IR did not attend. Data in c, d [95% CI for mean slope of all curves].

Primary objective of this case-control study was to study disease mechanisms associated with overweight and type 2 diabetes with focus on insulin resistance mechanisms in adipose tissue. We recruited 147 volunteers by newspaper listings for the MD-Lipolysis study at the Wallenberg Laboratory, Sahlgrenska University Hospital in Gothenburg. Initial contact was by telephone, and individuals likely to fit inclusion criteria were invited for a physical screening visit. In total, 67 participants were invited to attend the clinic for a screening visit with a physician. All eligible subjects (9 lean subjects (Lean), 9 insulin-resistant (IR) obese subjects (Obese-IR) and 9 obese subjects with type 2 diabetes (Obese-T2D) were white men and women (Fig. 1a, Table 1). The sample size was based on previous clinical microdialysis protocols from our group [28]. All subjects reported eating a mixed diet and being weight stable (±5 kg) for > 3 months before the screening visit. No subjects reported an “at-risk drinking of alcohol” pattern (> 7 drinks per week for women and > 14 drinks per week for men) nor were abstainers. Inclusion and exclusion criteria are briefly described in Fig. 1a and a detailed description is presented in Supplemental material. Medical history and concomitant medication for all subjects are shown in Table S1.

**Table 1:** Characteristics of subjects in MD-Lipolysis study.

	Lean	Obese-IR	Obese-T2D
Subject number (F/M)	6/3	5/4	5/4
Diabetes duration (years)	-	-	2 (2; 3)
Ethnicity (n)			
-Non-Hispanic White	9	9	9
Age (years)	63 (56; 66)	64 (59; 65)	59 (56; 64)
Weight (kg)	73 (62;76)	98 (93; 99)	101 (89; 106)
BMI (kg/m²)	23.9 (22.9; 24.3)	32.0 (31.4; 32.8)	34.0 (31.4; 35.6)
Waist (cm)	83 (76; 86)	109 (106; 115)	111 (106; 114)
Fat mass (kg)	19 (16; 24)	41 (29; 46)	35 (24; 45)
Systolic blood pressure (mmHg)	136 (115; 140)	133 (128; 144)	145 (135; 148)
Diastolic blood pressure (mmHg)	80 (70; 88)	81 (73; 89)	84 (81; 90)
B-HbA1c (mmol/mol)	32 (31; 35)	36 (33; 39)	50 (45; 59)
HOMA-IR*	1.2 (0.8; 1.4)	3.5 (2.5; 4.6)	2.4 (1.8; 4.9)
Matsuda index**	8.7 (6.8; 10.6)	4.5 (2.8; 5.3)	3.5 (2.5; 4.9)
S-HDL (mmol/l)	1.8 (1.5; 2.2)	1.3 (1.1; 1.5)	1.2 (0.9; 1.8)
S-LDL (mmol/l)	3.9 (3.1; 4.2)	4.2 (3.1; 4.6)	3.2 (2.8; 3.5)
S-Triglycerides (mmol/l)	0.7 (0.6; 1.0)	1.3 (1.2; 1.9)	1.9 (1.1; 2.4)
S-ALT (μkat/l)	0.33 (0.27; 0.39)	0.44 (0.37; 0.61)	0.75 (0.47; 1.02)
S-AST (μkat/l)	0.38 (0.31; 0.49)	0.36 (0.33; 0.43)	0.45 (0.41; 0.64)
S-Creatinine (μmol/l)	76 (70; 87)	88 (86; 99)	68 (65; 74)
U-Albumin/Creatinine (g/mol)	0.3 (0.3; 0.5)	0.7 (0.6; 1.6)	0.4 (0.3; 2.3)

ALT: alanine aminotransferase; AST: aspartate aminotransferase; BMI: body mass index; F: female;

HDL: high-density lipoprotein; HOMA-IR: homeostatic model assessment for insulin resistance;

LDL: low-density lipoprotein; M: male.

HbA1c, reference value: 31-46 mmol/mol; U-Albumin/Creatinine reference value: <3.0 g/mol.

*HOMA-IR is presented from Visit-2 for obese-T2D as insulin was not measured in this group at Visit-1.

**Data from the oral glucose tolerance test at Visit-2.

Continuous data are presented as median (25; 75-percentile), categorical data as n.

#### 2.1.2 Study protocol

All subjects in the MD-Lipolysis study were scheduled for 3 visits and arrived at the laboratory after fasting overnight (no food intake after 10 pm) (Fig. 1b). At Visit-1 a physician interviewed subjects for their medical history and current lifestyle and a physical examination was performed. An oral glucose tolerance test (OGTT, 75 g) was performed on non-diabetic subjects (lean, obese-IR) and a blood screen on all subjects (described under Laboratory procedures). Eligible subjects were scheduled for Visit-2, where an OGTT combined with microdialysis and subcutaneous blood flow measurement by ^133^Xenon clearance were performed. A subcutaneous needle biopsy and measurement of endothelial function were performed at Visit-3. Prior to Visit-2 and -3 subjects were asked to abstain from taking oral hypoglycemic agents, anti-hypertensive or lipid-lowering medication for 10 days (Fig. 1b). Investigators responsible for conducting the tests at Visit 1-3 did not perform the statistical analyses but participated in data interpretation.

#### 2.1.3 Study procedures

*Anthropometric measurements* – Subjects underwent measurements of height, weight, waist and hip once at Visit-1 (screening). Blood pressure, pulse rate and body temperature were registered and blood samples (after overnight fasting) were collected. Body fat (kg) was measured by bioimpedance using a BIA 101 according to the manufacturer's instructions (Akern, SMT Medical GmbH, Germany). Physical activity level was scored from 1–4 [29] (Supplemental Material).

*Adipose tissue microdialysis* - Abdominal subcutaneous microdialysis was conducted while the subject was in the supine position in a room kept at a stable temperature of ca 25 °C. Start of baseline measurements began 45 min after microdialysis catheter insertion. During sample collection, the right forearm was kept heated with an electric heating pad (OBH Nordica, Sundbyberg, Stockholm, Sweden) to arterialize venous blood in the distal forearm [28]. Blood samples and dialysates were collected every 15–30 min for 5 h. After 60 min of baseline measurements an OGTT (75 g) was performed with participants in a semisupine position. Subcutaneous microdialysis was then monitored for 3 h before ending measurement of metabolism in situ and insulin kinetics. An additional hour of dialysate collection and blood sampling to get three time points for inulin measurements was also performed, for a total of 4 h of sample collection (Supplemental Material).

*Subcutaneous microdialysis of interstitial glycerol, lactate and glucose* - Catheters for metabolite sampling were custom made glued to hollow semi-permeable fibers of hemicellulose (Cuprophane, molecular cut off 3 kDa, 0.25 × 30 mm, Gambro AB, Lund, Sweden) as previously described by our laboratory [30]. The catheters were inserted in parallel ca 30 mm apart in the periumbilical area and perfused with isotonic saline with addition of 1.5 mmol/l glucose, 25 µmol/l glycerol and 500 µmol/l lactate at a rate of 2.5 μl/min using a CMA 100 microinjection pump (MDialysis, Stockholm, Sweden) (Supplemental Material).

*Subcutaneous microdialysis of interstitial insulin* - Similarly, two hand-made microdialysis catheters used for protein sampling were inserted on the left-hand side of the abdomen [31]. The catheter was produced from a polyethylene membrane (Asahi, *Asahi* Kasei Medical Co, Japan) with an ID of 340 µm, an OD of 440 µm, a pore size of 0.3 µm and a 3 MDa molecular cut off. The tubing at the inlet of the catheter was connected to a syringe placed in a CMA 100 microinjection pump and perfused at a rate of 2.5 µl/min. Inulin was used as a reference substance for assessment of recovery for insulin [32] (Supplemental Material).

*Adipose tissue blood flow measurements by ^133^Xenon-clearance* - 4-6 MBq of ^133^Xenon gas was injected on both sides of the abdominal subcutaneous adipose tissue as previously described [28]. The activity of ^133^Xenon at the injection sites was registered in 30 s intervals using a GMS 411 Medscint (John Caunt Scientific, Lancashire, England) (Supplemental Material).

*Glycerol release* - According to Fick's principle, the release of a substance from any tissue is proportional to the blood flow and changes in the substance concentration. Thereby, glycerol release can be calculated as previously shown [28] (Supplemental Material).

*HOMA-IR and Matsuda Index* – Established assessments for approximation of insulin resistance [33,34] (Supplemental Material).

*EndoPAT measurement* - Peripheral endothelial function was determined by assessing peripheral arterial tonometry with the EndoPAT 2000 device (Itamar Medical, Caesarea, Israel). The device automatically calculated reactive hyperemia index and a ratio between baseline recordings and post-occlusion recordings [35] (Supplemental Material).

*Adipose tissue biopsy collection* - A subcutaneous needle biopsy was aspirated from the periumbilical region after subcutaneous administration of an anesthetic blockade (Carbocain 10 mg/ml without adrenaline, Aspen Nordic, Ballerup, Denmark) 5 min prior to biopsy [36]. Adipose tissue was snap-frozen in liquid nitrogen and some fresh tissue was used for measurement of adipocyte size (Supplemental Material).

*Adipocyte isolation and measurement of adipocyte size and number* – The procedures have been described in earlier papers from our laboratory [36]. This enabled the assessment of glycerol release per 100 g adipose tissue, glycerol release per 10^4^ adipocytes and surface area of 10^4^ adipocytes (Supplemental Material).

#### 2.1.4 Laboratory procedures

*Plasma glucose, plasma FFA and blood tests* - Plasma glucose measurement during the OGTT (Visit-2, arterialized venous plasma from the forearm), and in the morning at Visit-1 and Visit-3 (capillary plasma glucose) was analysed with HemoCue® Glucose 201^+^ (Hemocue AB, Ängelholm, Sweden, estimated mean coefficient of variation (CV) of 2.4%). Plasma free fatty acid (FFA) analyses were performed with an enzymatic colorimetric method at the Department of Clinical Chemistry, Sahlgrenska University Hospital, Gothenburg, Sweden. The CV for the method is 4.0%. Other analyses performed at the accredited laboratory at Department of Clinical Chemistry, Sahlgrenska University Hospital were measurements of blood samples from the screening visit (Supplemental Material).

*Insulin and inulin measurements in dialysates, serum and plasma* - At Visit-1, serum insulin was analysed for the non-T2D participants at an accredited laboratory at the Department of Clinical Chemistry, Sahlgrenska University Hospital. All measurements of serum insulin during Visit-2 were performed at the Wallenberg laboratory with the Mercodia Insulin ELISA assay (Mercodia AB, Uppsala, Sweden). The intra-assay and in between-assay CV for this method were 3.4% and 4.5%, respectively. The detection limit of the Mercodia ELISA kit was 3 mU/l and the cross-reactivity with proinsulin was <0.01%. Dialysate insulin was measured using an ultrasensitive enzyme immunoassay method (Mercodia Ultrasensitive Insulin ELISA, Mercodia AB, Uppsala, Sweden). The intra-assay and in between-assay CV for this method were 5.3% and 6.0%, respectively. The detection limit of the Ultrasensitive Mercodia ELISA kit was 0.15 mU/l and the cross-reactivity with proinsulin was <0.01%. Dialysate and plasma inulin were measured by a photometric method. The intra-assay CV for inulin was estimated to be approximately 10%. We used the external reference technique with inulin [32] (Inutest®, 0.25 g/ml, Fresenius Kabi Austria GmbH, Linz, Austria) to determine recovery for insulin *in situ*. The recovery of insulin in subcutaneous adipose tissue was (4±3, 3±3, 4±2 %, mean±SD) in lean, obese-IR and obese-T2D participants, respectively.

*Glycerol, lactate and glucose measurements in dialysates and plasma* - Dialysate glycerol, lactate, glucose and plasma glycerol and lactate, were analysed with an enzymatic colorimetric method, sample volume 0.2 µl, using a CMA 600 Microdialysis analyser (MDialysis AB, Stockholm, Sweden) (CV of 2.0, 4.4 and 3.9% for measurements in dialysates, and 1.4, 10.4, 7.6% for measurements in plasma, for glucose, lactate and glycerol, respectively). The endogenous reference technique [37] includes urea and is used for calculation of recovery for glucose, lactate and glycerol, thereby enabling assessment of their absolute concentration in the interstitial fluid in subcutaneous adipose tissue. We assessed the relative recovery for every metabolite (glucose: 39±16, 25±7, 34±10%; lactate: 36±10, 22±5, 29±9%; glycerol: 42±11, 25±6, 33±10%) in lean, obese-IR and obese-T2D participants, respectively (Supplemental Material).

*Total RNA extraction and quantitative PCR gene-expression analysis* - RNA was obtained from adipose tissue by the guanidinium–thiocyanate extraction method. cDNA was prepared using the ImProm-II™ Reverse Transcriptase (Promega, A3803), and qPCR was performed using the commercial SsoAdvanced™ Universal SYBR® Green Supermix (Bio-Rad 172-5274). Primer sequences are listed in Supplemental material.

*RNA sequencing of adipose tissue* - RNA concentration was measured using Nanodrop. Quality of RNA was evaluated using Tapestation 2200 (Agilent). Library preparation was performed using TruSeq Stranded Total RNA Sample Preparation Kit with Ribo-Zero Gold (Illumina). The pool of 25 samples was sequencing twice on a NextSeq500 instrument (Illumina) with Nextseq500 Kit High Output V2 reagent (Illumina), read length 2*75, and loading concentration 1,45pM.

*RNA sequencing data analyses* - For every sample, at least 35 million reads were sequenced. Raw sequence files were subjected to quality control analysis using FastQC (v 1.3) (http://www.bioinformatics.babraham.ac.uk/projects/fastqc/, accessed on May 2020). Transcript quantification was conducted with STAR (v2.5.3a) [38] (assigned to a gene using the GENCODE annotation (v27) using the STAR function “quantMode GeneCounts”) mapped to the human genome version GRCh38 and with reference annotation.

Read counts generated by STAR were analysed by using DESeq2 package [39] for detecting genes that were differentially expressed. An adjusted p-value cut off of 0.05 was decided as threshold for detection of DEGs. Starting from the expression matrix, genes that were considered as differentially regulated, were analysed using hierarchical clustering method (Cluster 3.0, http://bonsai.hgc.jp/~mdehoon/software/cluster/software.htm, accessed on May 2020). Visualization of the clustering and heatmap of log2-normalized values were obtained using Java Treeview. To explore the high-dimensional property of the data we used Principal Component Analysis (PCA), as dimensionality reduction algorithm implemented in stats package. For the Functional annotations analyses we used Enrichr web tools, accessed on May 2020. The enriched annotation table results, obtained in the web site, were then downloaded to be processed and visualized using ggplot2 package.

#### 2.1.5 Datasets deposited

RNA-seq processed files from subcutaneous adipose tissue of lean, obese and obese T2D individuals were deposited with accession number GSE141432 at Gene Expression Omnibus. https://data.mendeley.com/datasets/x5z3kyhmd8/draft?a=588446aa-d62a-42a9-90b6-ef4be7ff1b7f, last accessed February 2021.

### 2.2 POEM study

#### 2.2.1 Study participants

The POEM study was designed to investigate metabolism in a cardiovascular context and to investigate intermediary metabolism in relation to insulin resistance and was performed at Uppsala University. In this study, the primary endpoint was to study the relationship between insulin and free fatty acids in this normal urban population of East Sweden. The population-based, Prospective study on Obesity, Energy, and Metabolism (POEM) recruited Caucasian males and females all aged 50 years by a random invitation by mail using official registers of the inhabitants of the city of Uppsala. The participants received an invitation one month following their 50th birthday. The whole POEM cohort has previously been described in detail and the sample size was calculated for the cardiovascular research questions [40]. In total 502 individuals participated in the POEM study (participation rate 25%), of whom 499 did not have missing data in key variables. Data on blood glucose and serum insulin were available whereas plasma C-peptide and plasma FFA of these individuals were measured for this study. The participants were investigated in the morning after an overnight fast. The staff responsible for phenotyping of the participants in Uppsala and the investigator who did the analyses of FFA and C-peptide at the Wallenberg Laboratory did not perform the final statistical analyses and interpretation of the results.

#### 2.2.2 Laboratory procedures

FFA were measured using a commercial ELISA kit (LabAssay NEFA, Wako Chemicals GmbH, Neuss, Germany, within assay variation of absorbance CV < 1.5 %). The assay falls within the range of ± 15% of a known standard concentration. Plasma C-peptide was measured by an ELISA kit (Mercodia, Uppsala, Sweden, within assay variation CV < 5%). Other procedures used in the POEM cohort have been described previously [40].

### 2.3 Statistics

GraphPad Prism 9.0.0 software (GraphPad Inc., USA) was used for statistical analysis. A *p*-value <0.05 was set to determine statistically significant differences. Data in the MD-Lipolysis study are presented as median and interquartile range for continuous variables and absolute number and percent in respective group for categorical variables. Changes in variables between visits were assessed by calculating the 95% confidence interval of the mean slope within each group. Group comparisons for continuous variables were tested with Mann-Whitney U-test. For data analysed over time, AUC is presented and mixed-effects models are also available in the Supplementary material. Correlations were tested with Spearman's ranking. Statistically significant differences in gene expression (*p* < 0.05) were adjusted for multiple testing by using the FDR/Benjamini-Hochberg method.

Data from the POEM cohort are presented for all groups as mean ± 95% confidence interval, stratified by sex. Influence of metabolic state and sex on all variables was determined by 2-way ANOVA, and comparisons with the control group were assessed with Dunnett's post-hoc test, also stratified by sex. For FFA measurements, Dunnett's post hoc test between Lean and ObOw-HI groups was also conducted without stratification, but adjusting for sex.

### 2.4 Ethics

The MD-Lipolysis study and amendments to this project were approved by the regional Ethical Review Board, Gothenburg and the Swedish Ethical Review Authority, Uppsala, Sweden (Dnr 844-12, T 844-13, T 073-14, T 474-15, T 967-15 and 2019-04911) and an informed signed consent was obtained from all subjects before any study-related procedure was commenced. Informed written consent was obtained by all participants before entering the POEM study and the institutional Review Board in Uppsala approved the study (Dnr 2009/057). All investigations were performed according to the Declaration of Helsinki.

### 2.5 Role of the funding source

Funding sources did not have any influence on data collection, analysis, or interpretation; trial design; patient recruitment; or any aspect pertinent to the study.

## 3 Results

### 3.1 MD-Lipolysis study design and characteristics of study subjects

To learn on the mechanisms of insulin resistance in obesity-driven type 2 diabetes three groups were investigated: 1) lean subjects with normal glycaemia (Lean); 2) obese subjects who are hyperinsulinemic and insulin resistant but that maintain a good glycaemic control (Obese-IR); and obese subjects with recently diagnosed type 2 diabetes (Obese-T2D). Volunteers were well matched for age and sex allowing for unbiased group-wise comparisons of metabolic variables (Fig. 1a).

Out of 67 examined individuals at the screening visit, 31 participants fulfilled the inclusion and exclusion criteria, four participants were excluded after protocol violation (Table S2), and 27 participants entered and completed the study. Nine “Obese-T2D” (five post-menopausal females and four males) with median age of 59 years, who were recently diagnosed with type 2 diabetes (less than 6 years), (Table 1); nine “Obese-IR” who were not diabetic but were hyperinsulinemic and matched to the Obese-T2D group for age, sex, menopausal status, BMI, and fat mass (Fig. 1a and Table 1); and nine “Lean” subjects, six postmenopausal females and three males, matched for age with the other groups (Fig. 1a and Table 1).

Medications were withdrawn ten days before subcutaneous microdialysis and the glucose tolerance test at fasting, and before collection of adipose tissue biopsies (Fig. 1b). HbA1c levels and body weight were measured at each visit, and values were overall stable (Fig. 1c-d).

### 3.2 Obese-IR dissociate circulating levels of insulin, glycerol, and FFA, from glycaemia

**Fig. 2:**
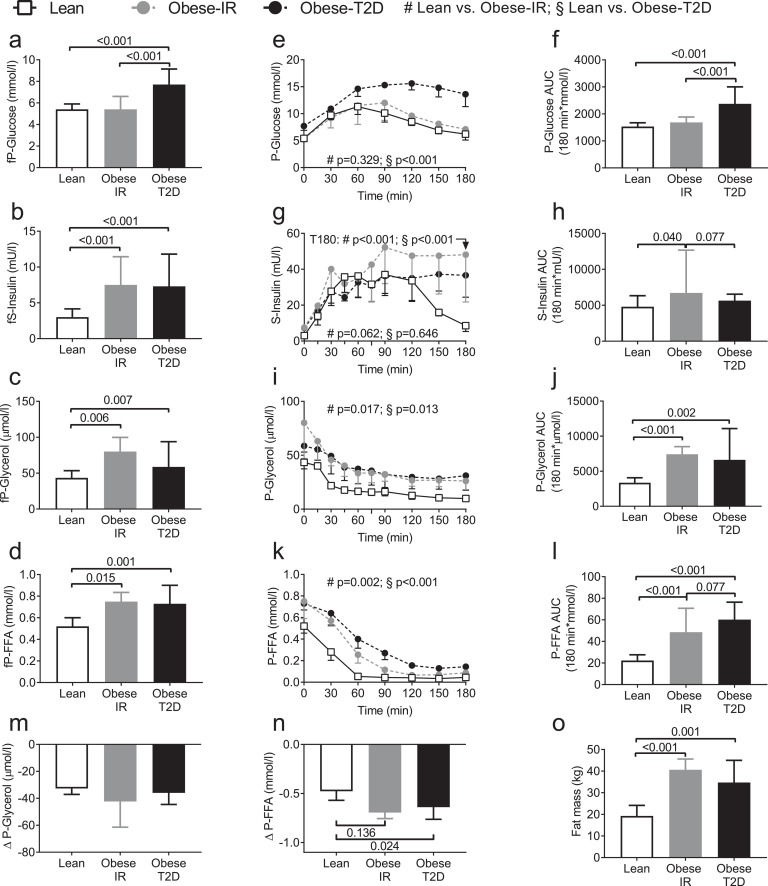
Circulating concentrations of insulin and metabolites in Lean, Obese-IR, and Obese-T2D subjects. Overnight fasting levels of: (a) plasma glucose (fP-Glucose); (b) serum insulin (fS-Insulin); (c) plasma glycerol (fP-Glycerol); and (d) plasma free fatty acids (fP-FFA). During an oral glucose tolerance test (OGTT) we measured the levels of: (e) plasma glucose (P-Glucose), and (f) the area under the curves (AUC) in e; (g) serum insulin (S-Insulin) and (h) AUC of g; (i) plasma glycerol (P-Glycerol), and (j) AUC of i; (k) plasma free fatty acids (P-FFA), and (l) AUC of k. Maximum decrease from the baseline value during the OGTT of the levels of (m) P-Glycerol, and (n) P-FFA. (o) Fat mass of study participants. Data are presented as median and error bars indicate interquartile range (IQR) for each group. n=9 in each group for all panels [Mann-Whitney U-test for bars and time 180 in figure (g), mixed-effects models for curves].

We measured fasting circulating levels of glucose, glycerol, FFA, and insulin, in Lean, Obese-IR, and Obese-T2D subjects. The results show that whereas fasting glucose levels of Obese-IR were similar to the one of Lean subjects, fasting insulin, FFA, and glycerol levels of Obese-IR were elevated to the same extent as of the one of Obese-T2D subjects (Fig. 2a-d). We next measured plasma glucose, insulin, glycerol, FFA and lactate levels during an oral glucose tolerance test (OGTT). Circulating glucose levels during the OGTT were essentially identical between Lean and Obese-IR subjects and, as expected, were markedly elevated in Obese-T2D subjects (Fig. 2e). Consistently, the area under the OGTT glucose curve of Obese-T2D was significantly larger than the one of Lean and Obese-IR subjects, which were similar among them (Fig. 2f). Insulin levels during the OGTT were higher in Obese-IR compared to the other groups, whereas insulin levels in obese-T2D were similar to Lean subjects until 120 minutes but did not normalise even at the end of the OGTT (Fig. 2g-h). Plasma glycerol levels of Obese-T2D and Obese-IR were virtually identical among them and equally elevated compared to Lean subjects (Fig. 2i-j). Plasma levels of FFA were highest in Obese-T2D, but FFA levels in Obese-IR were also statistically significantly elevated compared to Lean subjects (Fig. 2k-l). The maximal reduction of glycerol levels during OGTT was similar between groups, whereas maximal reduction of FFA levels was more pronounced in the obese subjects (Fig. 2m-n). Fat mass was drastically increased in both Obese-IR and Obese-T2D compared to Lean subjects (Fig. 2o).

Overall our results show that Obese-IR display fasting levels of insulin, glycerol and FFA which were elevated to a similar extent of the one of Obese-T2D, but showed fasting glycaemia and a glucose tolerance which were virtually identical to the one of Lean subjects. Furthermore, elevated levels of FFA and glycerol in the obese groups during OGTT were largely explained by higher levels at fasting (time point 0 min of OGTT) as the absolute drop of glycerol and FFA was not reduced in obese compared to lean subjects (Fig. 2m-n).

However, from these results it is not possible to conclude whether the fasting FFA and glycerol levels in Obese-IR were elevated compared to Lean subject because of an adipose tissue insulin resistance or because of their larger fat mass (Fig. 2o).

### 3.3 Circulating lactate increases progressively from Lean to Obese-IR to Obese-T2D subjects

Lactate, a powerful regulator of lipolysis which was implicated in the antilipolytic action of insulin [41], was also measured during the OGTT above. Fasting lactate levels increased progressively from Lean, to Obese-IR, and Obese-T2D subjects (Figure S1). During OGTT lactate levels were the highest in the Obese-T2D group whereas for Obese-IR lactate levels were relatively high at time 0 min, but by 45 min were similar to the one of Lean subjects (Figure S1). Overall fasting lactate increased progressively from Lean to Obese-IR to Obese-T2D without an obvious association with either FFA or glycerol levels.

### 3.4 Adipose tissue blood flow but not glycerol release is altered in obese subjects

**Fig. 3:**
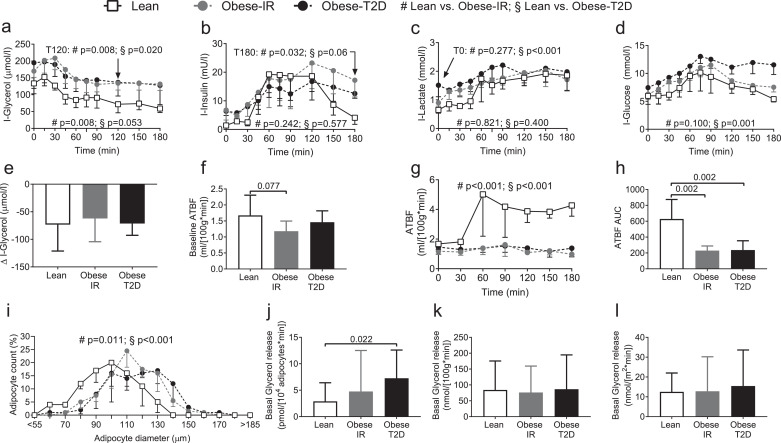
Measurements of abdominal subcutaneous adipose tissue function in Lean, Obese-IR, and Obese-T2D subjects. During the OGTT in Fig. 2 we collected dialysates of abdominal subcutaneous adipose tissue and measured the concentrations of: (a) Interstitial glycerol (I-Glycerol); (b) interstitial insulin (I-Insulin); (c) interstitial lactate (I-Lactate); and (d) interstitial glucose (I-Glucose). (e) Maximum decrease in I-Glycerol from the baseline value during the OGTT. (f) Basal subcutaneous adipose tissue blood flow (ATBF) measured by ^133^Xe-clearance technique. (g) ATBF during the OGTT; and (h) ATBF area under the curve (AUC) of g. Data are presented as median and error bars indicate interquartile range (IQR) for each group. (i) Adipocyte size distribution curves of abdominal subcutaneous adipose tissue biopsies from Lean; Obese-IR; and Obese-T2D volunteers. Basal glycerol release rates from abdominal subcutaneous adipose tissue were calculated per: (j) 10^4^ adipocytes; (k) 100 g of adipose tissue; and (l) adipocyte surface area. (a, c-e) *n* = 9 for Lean, *n* = 8 for Obese-IR and *n* = 9 for Obese-T2D. (b) *n* = 9 for Lean, Obese-IR and Obese-T2D. (f-i) *n* = 9 in Lean and Obese-IR, and *n* = 8 in Obese-T2D. (j-l) *n* = 9 for lean, *n* = 8 for obese-IR and n=8 for obese-T2D. [Mann-Whitney U-test for all bars and specific time points in curves a-c (mixed-effects models available in Supplementary Table 3), mixed-effects models for all time dependent curves (a-d, g). Mann-Whitney U-test for participant average cell size in (i)].

To better understand the mechanisms driving high levels of circulating FFA in the obese we measured abdominal subcutaneous adipose tissue blood flow by ^133^Xenon clearance, and glycerol, insulin, lactate, and glucose concentrations in the subcutaneous interstitial fluid using microdialysis technique after fasting overnight (time point 0 min), and following glucose ingestion (OGTT) [28,42,43]. Adipose tissue glycerol levels in Obese-IR were virtually identical to the one of Obese-T2D and were statistically significantly elevated compared to the one of Lean subjects (Figs. 3a and S2a). Local insulin levels were similar in Lean, Obese-IR and Obese-T2D subjects but interstitial insulin was more sustained in Obese-IR compared to lean subjects at 180 min (Figs. 3b and S2b). Adipose tissue interstitial concentrations of lactate were elevated in Obese-T2D compared to lean subjects at time 0 min of OGTT, but were similar between groups at later time-points (Figs. 3c and S2c). Glucose levels in the interstitial fluid of abdominal subcutaneous fat during OGTT were highest in Obese-T2D compared to the other groups (Figs. 3d and S2d). The maximal reduction of interstitial glycerol levels during OGTT was similar between groups (Fig. 3e), indicating that higher glycerol levels observed in the obese subjects is mostly due to the higher basal levels and not to a defective response to the glucose load (Figs. 3a and 3e).

Adipose tissue blood flow showed a trend toward reduced basal blood flow in obese compared to lean subjects (Fig. 3f), but this difference was not statistically significant. However, the Lean group showed a dynamic increase in local blood flow in response to the oral glucose load, which was absent in the obese individuals (Fig. 3g and h). We have also observed a reduced blood flow response by EndoPAT analysis and insulin appearance rate was significantly reduced in Obese-T2D compared to Lean subjects (Figure S2e –g).

Adipocyte size distribution was also measured in needle biopsies from the same fat pads analysed by microdialysis (abdominal subcutaneous) and compared to Lean subjects, adipocyte size distribution curves of Obese-IR and Obese-T2D subjects were significantly and progressively shifted toward larger sizes (Fig. 3i).

Measurement of basal glycerol release rates in subcutaneous abdominal fat showed that glycerol release is statistically significantly higher in Obese-T2D only when expressed per number of adipocytes (Fig. 3j). Indeed, when expressed per fat mass or per surface of adipocyte glycerol release rate was not statistically significantly elevated in Obese-T2D or Obese-IR compared to Lean subjects (Fig. 3k-l). Differences in glycerol concentrations between subcutaneous adipose tissue interstitial fluids and arterialized blood were also similar between groups (Figure S2h). It is concluded that lipolysis assessed by glycerol release rates in Obese-IR is overall similar to the one of Lean subjects. The non-statistically significant trend for higher glycerol release rates observed in Obese-IR compared to Lean subject (Fig. 3j) was expected, as adipocytes of Obese-IR are larger than the one of Lean individuals (Fig. 3i).

These results are consistent with the idea that the elevated FFA levels observed in Obese-IR subjects with high fasting insulin are mostly consequent to their increased fat mass, since glycerol release rates per fat mass, or adipocyte surface, and suppression of lipolysis by glucose in Obese-IR were overall similar to Lean subjects.

### 3.5 Metabolic inflammation in lean, Obese-IR and Obese-T2D subjects

We measured mRNA levels of markers of inflammation in biopsies from abdominal subcutaneous adipose tissue by real-time PCR, and systemic markers of inflammation in Lean, Obese-IR, and Obese-T2D subjects. mRNA abundance of macrophage markers and markers of macrophage activation and inflammation were elevated in both obese groups compared to Lean subjects, but to a higher extent in the Obese-T2D group (Figure S3a). Circulating levels of C-reactive protein (CRP) were also elevated in obese groups, and Obese-T2D showed also a small but statistically significant increase in circulating leukocytes compared to Lean subjects (Figure S3b-c). Altogether, obese subjects showed elevated levels of markers of adipose tissue and systemic inflammation which were most pronounced in the Obese-T2D.

### 3.6 Relationship between subcutaneous interstitial glycerol levels and fasting Insulin, adipocyte size, and inflammatory markers

To achieve a better understanding of the interactions between lipolysis and other factors in obese subjects we have investigated possible correlations between the area under the curves of abdominal subcutaneous adipose tissue interstitial glycerol levels during OGTT (I-Glycerol AUC (Figure S2a) and different parameters. A strong correlation was observed between I-Glycerol AUC, circulating fasting insulin levels and HOMA-IR (Figure S4a-b). Covariance analysis showed that I-Glycerol AUC correlated with HbA1c levels in Lean and obese-IR, whereas data from the Obese-T2D group were shifted on a curve at higher HbA1c levels compared to Lean and Obese-IR subjects and did not correlate with I-Glycerol AUC (Figure S4c). Furthermore, we have observed a solid correlation between I-Glycerol AUC, BMI, fat mass, adipocyte size, and markers of inflammation (Figure S4d-h).

Overall these correlations are consistent with a link between fatness and large adipocyte size, adipose tissue inflammation, adipose tissue lipolysis output and hyperinsulinemia, and further support the dissociation between lipolysis and glycaemic control.

### 3.7 RNA sequencing analysis of adipose tissue from Lean, Obese-IR and Obese-T2D subjects

**Fig. 4:**
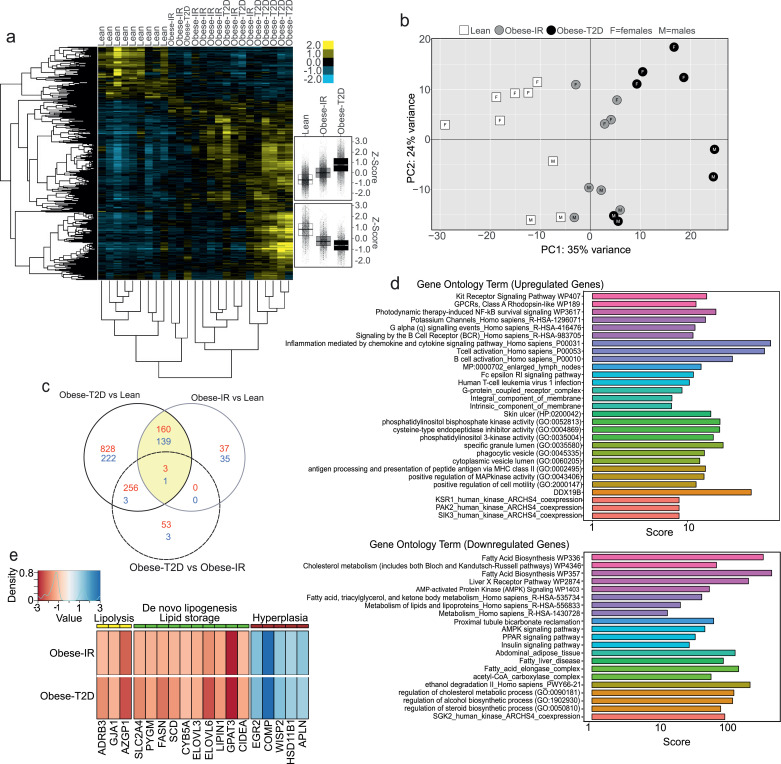
Adipose tissue RNA sequencing analysis in the MD-Lipolysis study. (a) Heat map showing the differentially expressed genes (log Fold change). Two main clusters of genes were obtained: one of genes upregulated and one of genes downregulated in the Obese groups. In the right panel, a box plot of these two gene clusters in the three categories [variance stabilizing transformation, FDR/Benjamini-Hochberg]. (b) Principal component analysis (PCA) of gene expression counts (normalized as in a). Groups and sex of participant are indicated. (c) Venn diagram of genes differentially expressed in the three comparisons. The number of differentially genes for each subgroup is indicated in red for downregulated and blue for upregulated. (d) Bar plots graph of the enrichment of ontology terms based on the combined score calculated by the EnrichR web tool. A selection of no more than the first five elements for each gene set databases is shown. Colour indicate the gene set database in Supplemental Material Figure 5a. All terms have multiple tests correction adjusted p-value less than 0.05 [FDR/Benjamini-Hochberg]. (e) Heat map of genes selected by literature search of all the core differentially expressed obesity genes identified by the Venn diagram (yellow area in c).

To further learn on the changes in adipose tissue gene-expression during the progression of obesity-driven diabetes we performed genome-wide RNA sequencing analysis of RNA preparations from abdominal subcutaneous adipose tissues of Lean, Obese-IR, and Obese-T2D subjects. Heat-map and box-plot analyses of differentially expressed genes showed a progressive change of gene-expression program from Lean to Obese-IR and to Obese-T2D (Fig. 4a). Indeed, both obese groups showed a similar pattern of gene-expression changes compared to Lean, but these changes were most pronounced in Obese-T2D individuals (Fig. 4a). Principal component analysis shows that principal component 1 (PC1) and principal component 2 (PC2) could explain most of data variability, with 35% of variance associated to PC1 and 24% of variance associated with PC2 (Fig. 4b). Along the PC1 axis it can be appreciated an obvious separation of datasets from Lean and obese groups, with Lean and Obese-T2D at the two extremes, while data from Obese-IR are in between these two groups (Fig. 4b). Hence, PC1 describes the progression from Lean to Obese-IR and to Obese-T2D. These three groups were not segregated along PC2, which instead identified the sex of the participants with perfect accuracy, as all the females showed positive values whereas all males showed negative values, independently from their adiposity or diabetes status (Figure 4b). This graph also indicates that there is no obvious effect of sex on the separation of the three groups along PC1. Consistently, comparison of males versus females showed a distinct set of differentially expressed genes than the one found from the comparison of Lean, Obese-IR, and Obese-T2D (Figs. 4 and S5).

Analysis of datasets from Lean, Obese-IR, and Obese-T2D individuals by Venn diagram identified a core of 303 genes which were differentially expressed in both obese groups against Lean individuals (Fig. 4c highlighted surface). Because circulating fasting FFA and glycerol levels (Figs. 2c-d) and interstitial glycerol levels in the analysed subcutaneous fat (Fig. 3a) were elevated in both Obese-IR and Obese-T2D to a similar extent compared to Lean individuals, we reasoned that the information concerning the control of fasting FFA and glycerol levels must be found in this common core of 303 differentially expressed genes. Unbiased gene ontology (GO) analysis identified several GO terms related to inflammation, immunity, and chemokine signalling among upregulated genes; whereas among the downregulated genes we found several GO terms related to de-novo lipogenesis and lipid synthesis (Fig. 4d). To achieve the best possible understanding of the mechanisms at the basis of the elevated FFA and on the metabolic response to obesity we performed an extensive literature search for each one of these 303 core obesity genes. The most upregulated genes were either genes abundantly expressed in leukocytes or genes implicated in leukocyte recruitment and inflammation whose levels were highest in the Obese-T2D group. No gene with an obvious implication in lipolysis was found among the genes upregulated in the obese groups. However, we have found that at least five genes with a demonstrated role in adipose tissue hyperplasia were upregulated in both obese groups compared to Lean individuals: Early growth response 2 (EGR2) also known as Krox20 [44]; cartilage oligomeric matrix protein (COMP) [45]; WNT1 inducible signalling pathway protein 2 (WISP2) [46]; hydroxysteroid 11-beta dehydrogenase 1(HSD11B1) [47]; and apelin (APLN) [48] (Fig. 4e). Among the genes whose mRNA levels were downregulated in the obese groups a most apparent effect was found on de-novo lipogenesis, as several genes directly implicated in lipid synthesis and glucose metabolic pathways to lipid synthesis were downregulated in the obese groups compared to Lean individuals. Among these genes: SLC2A4, encoding the glucose transporter GLUT4; PYGM, encoding glycogen phosphorylase; FASN, encoding fatty acid synthase; SCD, encoding stearoyl-CoA desaturase; CYB5A, encoding for cytochrome b5 type A, which is required for SCD activity; ELOVL3 and ELOVL6, encoding fatty acids elongases; GPAT3, which mediate the synthesis of lysophosphatidic acid; LIPIN1, an essential enzyme in the synthesis of diacylglycerol; and CIDEA a protein known to promote triglycerides storage by inhibiting basal lipolysis (Fig. 4e). However, the most revealing information was that mRNA levels of the β3 adrenergic receptor (ADRB3); gap junction protein alpha 1 (GJA1); and alpha-2-glycoprotein 1, zinc-binding (AZGP1), were all downregulated in obese individuals compared to Lean (Fig. 4e). Indeed, ADRB3 and GJA1 are essential for β-adrenergic signalling and lipolysis driven by catecholamine [49,50]; while AZGP1 is a potent promoter of β-adrenergic driven lipolysis [51].

Overall these results confirm the progression of adipose tissue inflammation from Lean to Obese-IR and Obese-T2D and indicate an upregulation of genes with a demonstrated role in hyperplasia, and downregulation of genes playing a key role in de-novo lipogenesis and β-adrenergic driven lipolysis. This observation is consistent with the idea that in obesity the adipose tissue undergoes an adaptive response restraining excessive FFA levels and adipocyte hypertrophic growth.

### 3.8 POEM study design and characteristics of study participants

**Fig. 5:**
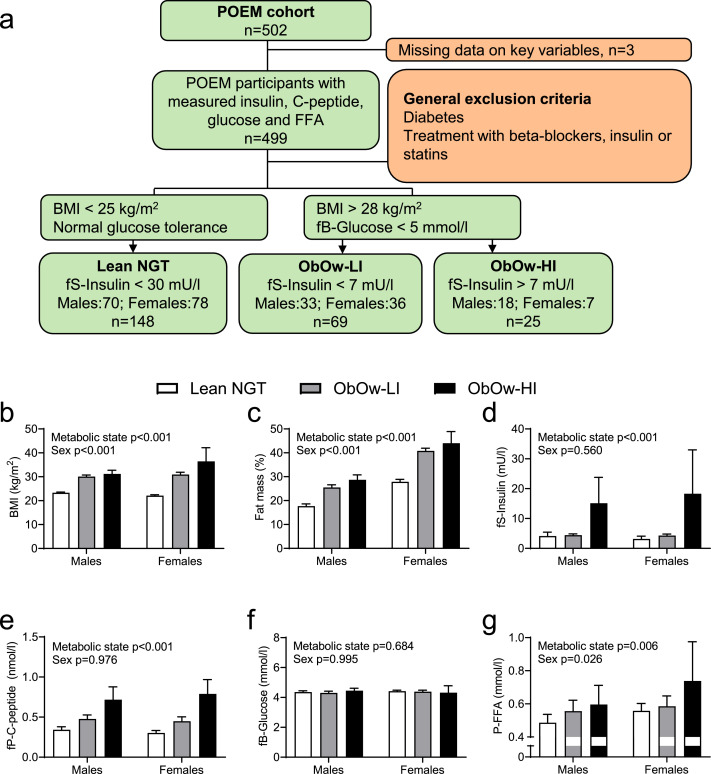
Study design and results on metabolic measurements from the POEM cohort. (a) Illustration of the volunteer selection procedure from the POEM study by specific inclusion (green) and exclusion (red) criteria, which defined the experimental groups: Lean NGT: lean normal glucose tolerance (lean insulin sensitive); ObOw-LI: obese-overweight normoglycaemic, with low insulin levels (obese-overweight insulin sensitive); and ObOw-HI: obese-overweight normoglycaemic, with high insulin levels (obese-overweight insulin resistant). Lean NGT were compared to ObOw-LI and ObOw-HI for: (b) body mass index (BMI); (c) fat mass; (d) fasting serum insulin levels; (e) fasting C-peptide levels; (f) fasting blood glucose levels; (g) fasting plasma FFA levels. n=148 for Lean NGT; n=69 for ObOw-LI; n=25 for ObOw-HI. Data are presented as mean ± 95% confidence intervals [two-way ANOVA for sex and metabolic group].

As the MD-Lipolysis is a case-control study performed on a limited number of subjects we decided to further investigate the association between circulating FFA levels and insulin secretion in obese and overweight (ObOw) subjects with normal glycaemic control from the population study POEM [40]. To Investigate the association between circulating FFA levels and insulin secretion in obese and overweight subjects with normal glycaemic control we measured fasting plasma FFA levels and C-peptide in 499 subjects from the POEM cohort for which data on fasting serum insulin levels; blood glucose levels; body mass index (BMI) and fat mass were available. All POEM participants are age-matched and were 50-years old (Table 2). Because our focus was on the initiation of insulin resistance and hyperinsulinemia in subjects with increased fatness but normal blood glucose, we excluded participants who were diabetic patients or lean participants who had fasting serum insulin levels above 30 mU/l; and participants who used beta-blockers, insulin, or statins. Confounders for our results not included in our analyses were alcohol intake, dietary habits, physical activity and socio-economic status of the participants in POEM. 148 Lean NGT (normal glucose tolerance) individuals (78 females and 70 males) were defined by a BMI < 25 kg/m^2^; whereas obese-overweight individuals (ObOw) with normal fasting glucose were defined with a BMI > 28 kg/m^2^ and fasting blood glucose < 5 mmol/l. Finally, ObOw individuals were divided into ObOw-LI (low insulinemia) showing insulin levels below 7 mU/l (36 females, 33 males); and ObOw-HI (high insulinemia) with insulin levels above 7 mU/l (7 females, 18 males) Fig. 5a and Table 2. The threshold of 7 mU/l was chosen to select the upper quartile of ObOw participants for insulin levels. To take in consideration the effects of sex, we used two-way ANOVA for sex-adjusted comparisons of: fasting levels of insulin, C-peptide, glucose, FFA, fat mass and BMI, in Lean-NGT; ObOw-LI; and ObOw-HI subjects (Fig. 5 b-g).

**Table 2:** Characteristics of subjects in the POEM cohort

	Lean NGT	ObOw-LI	ObOw-HI
Subject number (F/M)	78/70	36/33	7/18
Age (years)	50 ± 0	50 ± 0	50 ± 0
Weight (kg)	69 ± 10	92 ± 11	98 ± 13
BMI (kg/m²)	22.7 ± 1.6	30.5 ± 2.4	32.7 ± 4.7
Waist (cm)	84 ± 6	102 ± 7	107 ± 8
Fat mass (kg)	16 ± 4	30 ± 7	33 ± 11
Systolic blood pressure (mmHg)	117 ± 16	128 ± 20	127 ± 18
Diastolic blood pressure (mmHg)	75 ± 9	82 ± 12	83 ± 10
HOMA-IR	0.8 ± 1.0	0.9 ± 0.3	3.6 ± 3.8
S-HDL (mmol/l)	1.5 ± 0.4	1.3 ± 0.3	1,1 ± 0,2
S-LDL (mmol/l)	3,3 ± 0,9	3,6 ± 0,8	3.4 ± 0.9
S-Triglycerides (mmol/l)	0.9 ± 0.4	1.2 ± 0.6	2.4 ± 2.7
S-Creatinine (μmol/l)	77 ± 13	75 ± 12	77 ± 12
Current smoker (n, %)	13 (9)	8 (12)	4 (16)
Regular menses (n, %)	32 (22)	12 (17)	4 (16)
Hypertension (n, %)	6 (4)	1 (1)	2 (8)
Myocardial infarction (n, %)	0 (0)	0 (0)	1 (4)
Asthma or bronchitis (n, %)	10 (7)	7 (10)	4 (16)

BMI: body mass index; F: female; HDL: high-density lipoprotein; HOMA-IR: homeostatic model assessment for insulin resistance; LDL: low-density lipoprotein; M: male.

Continuous data are presented as mean (SD), categorical data as n (%).

### 3.9 Circulating FFA levels are elevated in ObOw with normal glucose and high insulin

Compared to Lean-NGT subjects, ObOw-LI and ObOw-HI subjects showed substantially increased fat mass (Fig. 5b,c). ObOw-HI showed also elevated fasting levels of insulin and C-peptide (Fig. 5d,e), whereas fasting glycaemia was virtually identical between groups (Fig. 5f). There was a clear effect of sex and metabolic state on circulating FFA levels, with females having higher FFA levels than males and ObOw-HI showing the highest levels of FFA among the three metabolic groups (Fig. 5g).

It is worth noting that compared to Lean-NGT, insulin levels increased only in ObOw-HI and not in ObOw-LI subjects, but FFA levels increased gradually from Lean-NGT, to ObOw-LI, and ObOw-HI (compare Fig. 5d with 5g Males). However, the levels of C-peptide, which is a better marker for insulin secretion than peripheral insulin levels, also increased gradually from Lean-NGT, to ObOw-LI, and ObOw-HI, similarly to FFA levels (compare Fig. 5e with 5g). To adjust for multiple comparisons, we also did a sex stratified Dunnett's post-hoc test in the POEM cohort (Table 3). Consequent to the selection criteria, ObOw-HI presented higher BMI, fat mass, fasting insulin and C-peptide in both males and females compared to Lean NGT (*p* < 0.001 for all comparisons), while there was no difference in fasting glucose (*p* = 0.474 and *p* = 0.682). Fasting FFA was statistically significantly higher in ObOw-HI in females (*p* = 0.048) and presented a similar trend in males (*p* = 0.079) compared to Lean NGT. Comparing ObOw-LI to Lean NGT also showed higher BMI, fat mass and C-peptide in both sexes (*p* < 0.001 for all comparisons). Furthermore, both groups presented similar glucose and insulin levels. Interestingly, FFA levels were also similar between the groups in both males and females (Table 3). Finally, the difference in FFA between Lean NGT and ObOw-HI was also analysed without stratification through a sex-adjusted Dunnett's post hoc test and was statistically significant (*p* < 0.05).

**Table 3:** Comparison of metabolic variables between Lean NGT, ObOw-LI and ObOw-HI during fasting in the POEM cohort

	Lean NGT vs. ObOw-LI				Lean NGT vs. ObOw-HI			
	Male		Female		Male		Female	
	Mean difference (CI)	p-value	Mean difference (CI)	p-value	Mean difference (CI)	p-value	Mean difference (CI)	p-value
BMI (kg/m²)	-6.8 (-7.8; -5.7)	<0.001	-8.9 (-9.8; -7.9)	<0.001	-7.9 (-9.2; -6.6)	<0.001	-14.3 (-16.3; -12.4)	<0.001
Fat mass (%)	-7.8 (-9.7; -5.8)	<0.001	-13.0 (-14.8; -11.1)	<0.001	-11.0 (-13.4; -8.5)	<0.001	-16.2 (-19.8; -12.6)	<0.001
fS-Insulin (mU/l)	-0.3 (-3.4; 2.8)	0.971	-1.1 (-4.1; 1.8)	0.630	-11.0 (-14.9; -7.1)	<0.001	-15.1 (-21.0; -9.3)	<0.001
fP-C-peptide (nmol/l)	-0.13 (-0.21; -0.06)	<0.001	-0.15 (-0.22; -0.07)	<0.001	-0.37 (-0.47; -0.28)	<0.001	-0.49 (-0.64; -0.34)	<0.001
fB-Glucose (mmol/l)	0.0 (-0.1; 0.2)	0.739	0.0 (-0.1; 0.2)	0.832	0.0 (-0.3; 0.1)	0.474	0.1 (-0.2; 0.4)	0.682
fP-FFA (mmol/l)	-0.07 (-0.17; 0.03)	0.194	-0.03 (-0.12; 0.06)	0.748	-0.11 (-0.23; 0.01)	0.079	-0.18 (-0.36; 0.00)	0.048

BMI: body mass index; FFA: free fatty acids.

Results from sex-stratified Dunnett´s post hoc test following 2-way ANOVA presented in Fig. 5.

*P*-value < 0.05 is considered statistically significant.

Overall these results indicate that FFA are statistically significantly and consistently elevated specifically in subjects with increased fatness who are hyperinsulinemic but not in subjects showing similar adiposity but normal insulin levels. Because fasting glucose in ObOw-HI was virtually identical to Lean NGT, and high FFA levels were associated with high C-peptide abundance, these results are consistent with the concept that elevated FFA may be the major driver of hyperinsulinemia in these subjects.

## 4 Discussion

Our results from the MD-Lipolysis study and the POEM study are consistent with the idea that elevated FFA levels, and not glucose, are the major metabolic derangement driving fasting hyperinsulinemia in obese insulin-resistant individuals with normal glycaemic control. The fact that fasting hyperinsulinemia was associated with high FFA levels in presence of normal fasting glycaemia is also consistent with studies indicating that insulin controls hepatic glucose production mainly through its direct action in the hepatocyte [52,53] rather than by reducing circulating levels of FFA and glycerol driving hepatic glucose production [3,17,54,55].

A second major finding from the MD-Lipolysis study is that elevated FFA in Obese-IR can be largely explained by their increased adipose tissue mass, rather than by an overt and uncompensated adipose tissue insulin resistance causing high rates of lipolysis per gram of adipose tissue. Elevated FFA and glycerol levels observed in Obese-IR and Obese-T2D were largely due to increased basal levels (fasting levels before the glucose load). Elevated levels of FFA and glycerol in presence of hyperinsulinemia are typically regarded as a manifestation of adipose tissue insulin resistance. However, it is important to consider that obese subjects have a much larger adipose mass than lean subjects and therefore FFA and glycerol levels are expected to be elevated even if the rate of lipolysis per gram of adipose tissue of obese subjects is comparable to the one of lean insulin sensitive subjects [26]. Adipose tissue lipolysis rates of Obese-IR subjects were virtually identical to the one of Lean controls if expressed per mass of adipose tissue or per adipocyte surface, and therefore the elevated FFA and glycerol levels observed in Obese-IR subjects can be mostly explained by increased fat mass and not by an overt uncompensated resistance to the antilipolytic action of insulin.

A third original finding from the MD-Lipolysis Study is that adipose tissue from Obese-IR and Obese-T2D showed reduced mRNA abundance of the β3-adrenergic receptor (ADRB3), which drives catecholamine-induced lipolysis via cAMP signalling [50]; and of gap junction protein alpha 1 GJA1, also known as connexin 43, which propagates catecholamine signalling by allowing the diffusion of cAMP between connected adipocytes [49]. Furthermore, we observed reduced mRNA levels of Zinc-alpha2-glycoprotein (AZGP1), which was shown to enhance β-adrenergic driven lipolysis [51]. High-basal but low catecholamine-stimulated lipolysis, measured in-vitro in isolated adipocytes, is a hallmark of obesity which was associated with weight gain and loss of glucose homeostasis [56], [57], [58]. Our data indicate a possible mechanistic explanation for such deranged lipolysis: the increased basal lipolysis, measured in-vitro in isolated adipocytes, can be explained by reduced levels of CIDEA, a most potent negative regulator of basal lipolysis [59], whereas reduced levels of ADRB3; GJA1 - connexin 43; and AZGP1 provide a sound mechanistic explanation for the reduced catecholamine-stimulated lipolysis observed in obesity [57,58]. It was also shown that cultured adipocytes from obese subjects are resistant to insulin action on de-novo lipogenesis, and on suppression of lipolysis measured in vitro [60]. Adipose tissue of Obese-IR and Obese-T2D showed reduced mRNA levels of several genes playing an essential role in lipid synthesis, which can fully explain the obvious defect in insulin-stimulated de-novo lipogenesis measured in-vitro in adipocytes from obese subjects [60]. By contrast, our results on adipose tissue glycerol release rates do not support the idea of an overt and uncompensated defect of the antilipolytic action of insulin in Obese-IR in vivo. However, it should be considered that differently from insulin action on lipogenesis, maximal insulin action on lipolysis in isolated adipocytes is not affected by obesity [60], thus an efficient antilipolytic insulin action is expected to be observed in vivo whenever enough insulin is present to compensate for insulin resistance. The EC_50_ for the antilipolytic action of insulin measured in-vitro in adipocytes isolated from obese subject is in the femtomolar-subpicomolar range [60], whereas the insulin levels we measured in interstitial fluids of subcutaneous adipose tissue are in the picomolar-subnanomolar range. Thus, it is logical that Obese-IR subjects have not showed a statistically significant defect in the antilipolytic action of insulin, since enough insulin was present in the adipose tissue interstitial fluids to compensate for an eventual adipocyte insulin resistance.

The reduced expression of ADRB3, GJA1, and AZGP1 observed in adipose tissue of obese subjects is consistent with the idea that adipose tissue from obese actively controls its lipolysis output to avoid excessively high levels of plasma FFA [26]. The idea of an adaptive response to adipose tissue expansion and increased FFA levels during obesity is also consistent with the general gene expression signature and the changes in regional blood flow we observed. Reducing lipolysis in a condition of chronic positive energy balance is expected to avoid an excessive raise of circulating FFA and glycerol levels but also to promote adipocyte hypertrophy. Hence, the blunted adipose tissue blood flow response to glucose, reduced expression of lipogenic genes, and increased expression of genes implicated in hyperplasia observed in obese subjects, may serve to avoid excessive adipocyte hypertrophy. Further support to the idea that reduced β-adrenergic sensitivity and reduced de-novo lipogenesis may be part of an integrated homeostatic response to adipose tissue expansion can be derived from the fact that at least five genes implicated in lipid accumulation, which were downregulated in the obese groups, are known to be induced by β-adrenergic-cAMP signalling: solute carrier family 2 member 4 (SLC2A4) encoding the glucose transporter GLUT4 [61]; fatty acid synthase (FASN) [23]; stearoyl-CoA desaturase (SCD) [23,62]; ELOVL fatty acid elongase 3 (ELOVL3) [63]; cell death-inducing DFFA-like effector a (CIDEA) [23,64].

The MD-Lipolysis study has some limitations which should be considered. We measured glycerol release as marker of lipolysis in situ, but we do not have data on FFA re-esterification or FFA oxidation rates by skeletal muscle. Our data do not exclude that a possible contribution for reduced FFA re-esterification in adipose tissue or reduced FFA uptake in skeletal muscle could have a potential contribution to increased FFA levels in obesity. However, a major role for these mechanisms is difficult to reconcile with as the drop of circulating FFA levels observed in Obese-IR subjects during the OGTT appears to be even more pronounced than the one of glycerol. Furthermore, an overt and uncompensated adipose tissue insulin resistance is expected to significantly increase glycerol release rates normalized on mass of adipose tissue or on adipocyte surface area.

It is also important to consider that our microdialysis measurements are performed in abdominal subcutaneous adipose tissue, thereby these data may not be extrapolated to all fat depots. Yet, the abdominal subcutaneous adipose tissue is a major adipose depot in obese humans and thus, it is likely a major contributor to systemic levels of FFA [65]. In the MD-Lipolysis study we have not investigated obese subjects with low insulin and FFA levels, and it is possible that additional adaptive mechanisms to fatness are in place in these subjects. Furthermore, the relatively small number of participants in the MD-Lipolysis study makes it difficult to extrapolate to the general obese population our conclusion that hyperinsulinemia and insulin resistance may develop as part of an adaptive response to increased FFA levels in obesity. However, this limitation is largely addressed by our analysis of fasting levels of insulin; C-peptide; FFA; and glucose from participants of the population-based POEM study.

Our data from the POEM study indicate that FFA levels are elevated in subjects with increased adiposity and high insulin levels (ObOw-HI), but are not significantly and consistently elevated in subjects with similar adiposity and low insulin levels (ObOw-LI).

Because fasting glycaemia in ObOw-HI was identical to the one of Lean controls, these results are consistent with the idea that FFA play an important role in driving basal insulin secretion in insulin-resistant overweight individuals with normal glycaemic control. The association between fasting levels of FFA and insulin was less obvious in male subjects as, compared to Lean-NGT subjects, insulin was increased only in ObOw-HI whereas FFA levels increased progressively from Lean-NGT, to ObOw-LI, and ObOw-HI. However, we have found that the levels of C-peptide, which reflect insulin secretion better than insulin levels, also increased progressively from Lean-NGT, to ObOw-LI, and ObOw-HI.

A limitation of data from POEM study is that it focuses on fifty-year old Caucasian individuals and thus it will be important to investigate whether FFA are also specifically elevated in obese individuals with normal glucose control in different age groups and of different ethnicities to further extend and confirm our finding. Nonetheless, our results from the POEM study are consistent with the idea that FFA are a major candidate driving fasting insulin secretion and fasting hyperinsulinemia, which may initiate insulin resistance, especially in the liver. It is well established that, apart from some amino acids, cultured pancreatic β-cell secrete insulin in response to either elevated glucose or FFA and that FFA potentiate glucose stimulated insulin secretion [[8], [9], [10],^66^]. Glucose and FFA induced insulin secretion are also modulated by some hormones and neurotransmitters, but ultimately insulin is secreted in response to changes in the levels of one of these metabolites [66]. Although the role for FFA in the stimulation of insulin secretion in cultured β-cell is established, the in-vivo significance of FFA-driven insulin secretion is not completely understood. There is no linear correlation between FFA and insulin levels and FFA levels are not always associated with insulin secretion [67], [68], [69]. Acute elevation of FFA above endogenous levels via intralipid and heparin infusion does not stimulate insulin secretion [70], [71], [72], [73], [74]. Finally, short-term overfeeding can drive hyperinsulinemia while reducing FFA levels [75,76]. However, our results indicate that elevated FFA levels from adipose tissue lipolysis may be particularly important in driving fasting hyperinsulinemia in obese subjects. This view is supported by studies showing that in-vivo blockage of adipose tissue lipolysis using nicotinic acid reduces fasting insulin secretion, and that the effects of nicotinic acid on insulin secretion are blocked by restoring the FFA levels [10,77]. Furthermore, obese mice with a loss of function mutation on the FFA receptor GPR40 display reduced insulin levels at fasting [78].

Because endogenous production of glucose and FFA are potently suppressed by insulin, fasting insulin secretion by the pancreatic β-cell appear to be regulated by two feedback loops: the first loop is with the hepatocyte via glucose production, whereas the second loop is with the adipocyte via FFA release from lipolysis. In normal weight subjects, the FFA feedback loop to β-cell insulin secretion appear to be recruited after a prolonged fasting (eg. more than 24 h), whereas in obese individuals the effects of FFA on insulin secretion were observed already after an overnight fasting [79], [80], [81]. It follows that resistance to insulin action in glucose metabolism may, at least in part, be consequent to high fasting insulin levels, which may develop as part of a homeostatic response to high levels of FFA [2,[4], [5], [6], [7],^26^].

According to this view, obesity-driven diabetes could be seen as a failure of the adaptive response to adipose tissue expansion. A better understanding of this adaptive response may pave the way for the development of novel therapeutics for the treatment of obesity-driven diabetes and the metabolic syndrome.

## Declaration of Competing Interest

L.M.G holds an employment at AstraZeneca R&D. All other authors declare no competing interest.

## Appendix Supplementary materials

Image, application 1

Image, application 2

Image, application 3

Image, application 4

Image, application 5

Image, application 6
