# Supplementary material for: Hyperinsulinemia and insulin resistance in the obese may develop as part of a homeostatic response to elevated free fatty acids: A mechanistic case-control and a population-based cohort study
Source: eBioMedicine. 2021 Mar 9;65:103264. doi: 10.1016/j.ebiom.2021.103264 (PMC7992078; doi:10.1016/j.ebiom.2021.103264)
Supplement: Supplementary file 1 [file mmc1.docx]

**Supplemental material**

**List of reagents, software and primer sequences.**

| REAGENT or RESOURCE | SOURCE | IDENTIFIER |
| --- | --- | --- |
| Biological Samples |  |  |
| Human blood and dialysate samples from Lean, insulin-resistant obese (Obese-IR), and obese type 2 diabetes (Obese-T2D) subjects | This manuscript | N/A |
| Human white adipose tissue from Lean, Obese-IR, and Obese-T2D subjects | This manuscript | N/A |
| Chemicals, peptides, and recombinant proteins | | |
| Glucose powder | Apotek Produktion & Laboratorier | Cat#323188 |
| ¹³³Xenon | IDB Holland | Cat#8286.008 |
| Gibco™ Medium 199 (+ 4 % bovine albumin) | Thermo Fisher | Cat#11150059 |
| Collagenase | Sigma-Aldrich | Cat#C6885 |
| Inutest® 0.25 g/ml | Fresenius Kabi | Cat#LI2114262 |
| Guanidinium thiocyanate | Sigma-Aldrich | Cat#G6639 |
| Sarkosyl (N-lauroylsarcosine sodium salt) | Sigma-Aldrich | Cat#L5125 |
| Critical commercial assays | | |
| Mercodia insulin ELISA | Mercodia | Cat#10-1113-01 |
| Mercodia ultrasensitive insulin ELISA | Mercodia | Cat#10-1132-01 |
| LabAssay NEFA | Wako Chemicals GmbH | Cat#294-63601 |
| C-peptide ELISA | Mercodia | Cat#10-1136-01 |
| Calibrator A | M Dialysis AB | Cat#P000057 |
| Glucose reagent | M Dialysis AB | Cat#P000023 |
| Lactate reagent | M Dialysis AB | Cat#P000024 |
| Urea reagent | M Dialysis AB | Cat#P000026 |
| Glycerol reagent | M Dialysis AB | Cat#P000025 |
| ImProm-II™ Reverse Transcriptase | Promega | Cat#A3803 |
| SsoAdvanced™ Universal SYBR® Green Supermix (qPCR mix) | Bio Rad | Cat#172-5274 |
| Deposited data | | |
| RNAseq raw data | GEO | GSE141432  wxudkewkvzmvjoh |
| RNA sequencing tables | Mendeley | https://data.mendeley.com/datasets/x5z3kyhmd8/draft?a=588446aa-d62a-42a9-90b6-ef4be7ff1b7f |
| Software and algorithms | | |
| GraphPad Prism v 8.1.1 | GraphPad Software | RRID:SCR_002798 |
| CFX Manager software | Bio-Rad | RRID:SCR_017251 |
| GMS411 Mediscint gamma counter system | John Caunt Scientific Ltd | <http://www.johncaunt.com/products/gms411> |
| Oligonucleotides | | |
| GENE NAME | FORWARD PRIMER | REVERSE PRIMER |
| LRP-10 GenBank NM_014045 | GCAGTGCTCTTAGAAGTGCAG | CCTGATGGTGACAGTCTGTTC |
| CD68 GenBank NM_001251 | CTTCTCTCATTCCCCTATGGACA | GAAGGACACATTGTACTCCACC |
| CD11b GenBank NM_001145808 | ACAGCCTTGACCTTATGTCATGG | CATTGCGTTTTCAGTGTCCAA |
| CD14 GenBank NM_000591.3 | TCTCTGTCCCCACAAGTTCC | CCCGTCCAGTGTCAGGTTATC |
| MCP-1 (CCL2) GenBank NM_002982 | CAGCCAGATGCAATCAATGCC | TGGAATCCTGAACCCACTTCT |
| TNFa GenBank NM_000594 | TCCTTCAGACACCCTCAACC | AGGCCCCAGTTTGAATTCTT |
| IL-1b GenBank NM_000576 | TTCGACACATGGGATAACGAGG | TTTTTGCTGTGAGTCCCGGAG |
| MRC-1 GenBank NM_002438 | CTACAAGGGATCGGGTTTATGGA | TTGGCATTGCCTAGTAGCGTA |
| CD163 GenBank NM_004244.5 | TTTGTCAACTTGAGTCCCTTCAC | TCCCGCTACACTTGTTTTCAC |
| IL-10 GenBank NM_000572 | TCAAGGCGCATGTGAACTCC | GATGTCAAACTCACTCATGGCT |
| VCAM-1 GenBank NM_001199834 | GGGAAGATGGTCGTGATCCTT | TCTGGGGTGGTCTCGATTTTA |
| ICAM-1 GenBank NM_000201 | GCAGTCAACAGCTAAAACCTTCC | GCTGGCAGGACAAAGGTCTGG |
| eNOS GenBank NM_000603.4 | TGATGGCGAAGCGAGTGAAG | ACTCATCCATACACAGGACCC |

**METHODS**

**MD-Lipolysis study**

**Study participants**

For participation in the MD-Lipolysis study detailed inclusion criteria were age 50-70 years (M) and 54-70 years (F); no menstruations during the last year or follicle-stimulating hormone (FSH) > 26 mIU/l (F); BMI 18-25 kg/m^2^ in lean subjects and BMI 30-40 kg/m^2^ in obese-IR and obese-T2D subjects; fasting insulin ≥ 9 mU/l in obese-IR subjects; and known T2D duration ≤ 6 years. Detailed exclusion criteria were history of ischemic heart disease, heart failure (NYHA II-IV), atrial flutter, heart valve disease, cerebral infarction, transitory ischemic episodes, peripheral artery disease or hypertension requiring 3 anti-hypertensive medications or more; daily smoking and daily frequent use of smokeless tobacco not enabling the participant to suspend nicotine during the investigations scheduled for day 2 and 3 because of physical abstinence; treatment with beta-blockers; anti-diabetic treatment including insulin, glucagon like peptide-1 (GLP-1) analogues, thiazolidinediones and dipeptidyl peptidase-IV (DPP-IV) inhibitors; hematologic diseases such as anaemia, blood malignancies and bleeding disorders; bleeding tendency due to illness or medical treatment (e.g., acid salicylic acid (ASA)); other conditions making it hard to participate, e.g., mental illness, back pain or substance abuse; and for obese-T2D subjects, significant complications resulting from T2D including pre-proliferative or proliferative retinopathy, neuropathy and renal failure defined as glomerular filtration rate (GFR) < 60 ml/min/1.73 m^2^.

**Study protocol**

All obese-IR and lean subjects in the MD-Lipolysis study performed an OGTT at visit-1 (screening). Impaired glucose tolerance (IGT) was defined by a 2-hour capillary plasma glucose concentration at 8.9-12.1 mmol/l (2-hour glucose was 7.1±1.3 and 7.9±1.3 mmol/l in lean and obese-IR subjects, respectively). One lean and 1 obese-IR subject showed IGT after the OGTT. Impaired fasting glucose (IFG) was defined by a fasting overnight capillary plasma glucose at 6.1-6.9 mmol/l (1). There was no subject with IFG in the lean but 2 subjects with IFG in the obese-IR group. HOMA-IR at 2.5 was our approximate level chosen to indicate insulin resistance, as previously suggested (2). T2D diagnosis was based on self-reported medication of oral hypoglycemic agents and/or lifestyle treatment. Subjects having combination therapy including calcium channel blockers for their hypertension were allowed, as per the protocol, to restart this medication if blood pressure > 180/105 mmHg at check-up 3-4 days prior to Visit-2 and -3.

**Study procedures**

*Anthropometric measurements* - Height was measured to the nearest 0.5 cm using an ordinary height-measuring gauge. Body weight (BW) was measured on a flat scale to the nearest 0.1 kg (Seca GmbH, Hamburg, Germany) with the subjects wearing underwear and socks. These height and BW measurements were used to calculate BMI ([BW (kg)]/[height (m)]^2^). Waist and hip circumferences were assessed to the nearest centimeter using a non-elastic tape measure with the subject standing. The waist circumference was measured midway between the lowest rib and the iliac crest whereas the hip circumference was measured at the widest part of the buttocks. Waist-hip circumference ratio (WHR) was calculated from these measurements. An ear thermometer (ThermoScan, Braun 7, Kronberg, Germany) was used to estimate body temperature (ºC). Systolic and diastolic blood pressure (mm Hg) was measured in the left arm using a sphygmomanometer (OMRON 705 IT, Omron Electronics AB, Kista, Sweden), while the subject was in the supine position after ca 10 min rest. For documentation of blood pressure at baseline the mean of two measurements was calculated. Inactive lifestyle was scored as 1, whilst walking, gardening or transport cycling at least 4 hr every week was scored 2. Regular but moderate exercise such as jogging or gym training at least 2 hr every week was scored 3 and intensive exercise at least 2 hr every week was scored 4.

*Subcutaneous microdialysis of interstitial glycerol, lactate and glucose* - Catheters for metabolite sampling were custom made of inert polyethylene tubing (0.58 mm inner diameter (ID) and 0.96 mm outer diameter (OD)), (Smiths Medical^TM^ Portex^TM^ Fine Bore LDPE Tubing, Minneapolis, MN, USA) glued to hollow semi-permeable fibers of hemicellulose (Cuprophane, molecular cut off 3 kDa, 0.25 x 30 mm, Gambro AB, Lund, Sweden) using cyanoacrylate (LOCTITE, Henkel Norden AB, Bromma, Sweden) and sent for ethylenoxide sterilization (Sterigenics Belgium S.A., Petit-Rechain (Verviers), Belgium). The inlet lengths of the catheters were 7 cm and the outlet lengths were standardized to 5 cm (the collection sites of the dialysates). The inlets were connected by custom-made connectors (B Braun Melsungen, Germany) to 50-70 cm long polyethylene tubing, which in turn were connected to syringe pumps. Two hand-made microdialysis catheters used for metabolite sampling were inserted without anesthesia on the right-hand side of the abdomen approximately 7-10 cm lateral of the umbilicus. Briefly, a cannula (18G, 1.2 x 50 mm, KD Medical GmbH, Berlin, Germany) was inserted through the epidermis at an angle of ca 30°. The cannula was passed parallel with the skin at 3-5 mm depth for 30-35 mm before passing up through the skin again. The lumen of the cannula then guided the microdialysis catheter through the tissue. After insertion of the probe in the tissue the cannula was withdrawn similar to the insertion of a guide wire using the Seldinger technique.

*Subcutaneous microdialysis of interstitial insulin* - Similarly, two hand-made microdialysis catheters used for protein sampling were inserted without anesthesia on the left-hand side of the abdomen approximately 7-10 cm lateral of the umbilicus (3). The probes were manufactured in Germany and introduced at the laboratory through a collaboration with Prof. Martin Schmelz, Department of Anaesthesiology, University of Heidelberg. The catheter was produced from a polyethylene membrane (Asahi, *Asahi* Kasei Medical Co, Japan) with an ID of 340 µm, an OD of 440 µm, a pore size of 0.3 µm and a 3 MDa molecular cut off. The semi-permeable membrane was connected to a tubing with a larger diameter at the inlet of the catheter, enabling it to connect to a syringe pump. A thin needle attached at the outlet of the catheter was used for insertion of the catheter through the subcutis. The needle was inserted into the adipose tissue in a similar way as the cannula was inserted for interstitial metabolite sampling. The length of the catheter in situ was approximately 30 mm. After insertion, the needle was cut with a pair of scissors, leaving an outlet of approximately 15 mm of the membrane. A capillary tube (micropipette, 100 µl, made of borosilicate glass (A. Hartenstein GmbH, Würzburg, Germany)) was placed at an angle of ca 40º onto the outlet of the membrane in order to optimise sampling and reduce the degree of ultrafiltration. The tubing at the inlet of the catheter was connected to a syringe placed in a CMA 100 microinjection pump and perfused at a rate of 2.5 µl/min. The perfusion solution used in the Asahi catheters was isotonic saline with addition of 1% human albumin and 1.5 mmol/l glucose (Albumin Baxalta 200 g/l, Shire Sweden AB, Stockholm, Sweden) in order to get a similar colloid osmotic pressure inside the membrane as compared to the surrounding extracellular fluid. 1% albumin in isotonic saline was perfused through the catheters before starting the experiments in order to avoid molecules of interest sticking to the catheter system during the measurements. It is known that an infusion of the polysaccharide inulin leads to a full equilibration between plasma and the interstitial fluid, i.e. plasma inulin equals interstitial inulin. Inulin is therefore used as a reference substance for assessment of recovery for insulin (4). Thus, inulin was administered intravenously (Inutest^®^ 0.25 g/ml, Fresenius Kabi Austria GmbH, Linz, Austria) at a bolus dose of 0.2 ml/kg body weight (BW) followed by a continuous infusion of 24 ml/hr for 4 hr. All dialysates and blood tubes without gel were immediately put on ice. Blood tubes were centrifuged after approximately 30 min (4000 rpm, 2000*x*g, 10 min, Hettich Universal 320R, Andreas Hettich GmbH & Co. KG, Tuttlingen, Germany) and aliquots of plasma treated with EDTA, sodium fluoride, and heparin as well as serum and dialysates were immediately frozen at -20°C.

*Adipose tissue blood flow measurements by ^133^Xenon-clearance* - 4-6 MBq of ^133^Xenon dissolved in 0.1-0.2 ml of gas was taken out with the aid of a 1 ml syringe and a sterile cannula. The ^133^Xenon was injected slowly at a depth of ca 5 mm during 1 min on both sides of the abdominal subcutaneous adipose tissue ca 10 cm above the microdialysis catheters, for assessment of mean adipose tissue blood flow (ATBF) at baseline and during OGTT. After 60 min of equilibration, the activity of ^133^Xenon at the injection sites was registered in 30-second intervals using a GMS 411 Medscint (John Caunt Scientific, Lancashire, England) and blood flow was calculated using the following formula:

$$ATBF \left( \frac{mL}{100 g*min} \right)= (Linear reg(Ln (count ), time)*\lambda*100 (g)*60 (sec)$$

The tissue-blood partition coefficient of ^133^Xenon was set to 10.0 for lean and obese participants according to previous experiments (5). One obese-T2D subject has no ATBF value (poor quality of the clearance curve) and therefore lack estimation of glycerol release.

*Glycerol release* - According to Fick’s principle, the release of a substance from any tissue is proportional to the blood flow and changes in the substance concentration. Thereby, glycerol release can be calculated according to the following formulas (5):

$$Glycerol release=\left( V-A \right)*Adipose tissue blood flow (ATBF)*(1-EVF)$$

Where *(V-A)* is the difference in glycerol concentration between venous and arterial plasma, and EVF is the erythrocyte volume fraction. If unknown, (*V-A*) can be substituted with the formula:

$$\left( V-A \right)=\left( I-A \right)*(1-e^{\frac{-PS}{Q}})$$

Here, *I* is the interstitial concentration of glycerol, A is the glycerol concentration in arterialised venous plasma and *PS* is the permeability surface constant for glycerol and Q is plasma flow rate. PS for glycerol has been estimated to be ≈ 5. Combining these formulas, glycerol release can be calculated as follows:

$Glycerol release=\left( I-A \right)*(1-e^{\frac{-PS}{Q}})$ $*ATBF*(1-EVF)$

EVF was missing in one lean, one obese-IR and one obese-T2D subject and was therefore approximated by using the mean EVF value for the respective group. One obese-IR subject lack glycerol release data because no needle biopsy was taken in that subject. That subject developed a large hematoma after the microdialysis procedure and therefore we cancelled visit-3 and the needle biopsy.

*HOMA-IR and Matsuda Index* - HOMA-IR was calculated as:

$$HOMA-IR=\frac{(glucose*insulin)}{22.5}$$

Matsuda Index was calculated from time points 0, 30, 60, 90 and 120 min during an OGTT. Glucose at 30 min had to be imputated for calculations for one Lean, and insulin at 60 min had to be imputated for one Obese-IR. Matsuda Index was calculated as presented below. In POEM, blood glucose was converted to plasma glucose with a conversion factor of 1.12.

$$Matsuda index=\frac{10 000}{\sqrt{{Glucose}_{0}{*Insulin}_{0}*{Glucose}_{mean}{*Insulin}_{mean}}}$$

*EndoPAT measurement* - Designed finger probes were placed bilaterally on each index finger to assess digital volume changes before, during and after complete occlusion of blood to the test arm. In short, a blood pressure cuff was placed on the upper part of the dominant arm, while the other arm was used as a control. After 6 min of baseline recordings, occlusion was applied using the cuff during 5 min, then the pressure was released and post-occlusion recordings were measured during an additional 5 min. One obese-IR subject and one obese-T2D subject lack results from the EndoPAT measurement due to a cancelled visit-3 and a suboptimally performed study procedure. During the EndoPAT we obtained very low signal at 4.5-5.5 min post-occlusion in all studied groups and therefore we have few results the last minute during the investigation.

*Adipose tissue biopsy collection* - An anesthetic blockade using Carbocain 10 mg/ml without adrenaline (Aspen Nordic, Ballerup, Denmark) was administered 5 min prior to biopsy. Subcutaneous adipose tissue was then aspirated with a needle (14G, 2.1 x 80 mm, KD Medical GmbH, Berlin, Germany) and a 20cc syringe (Codan Medical Aps, Rödby, Denmark) from the periumbilical region. Adipose tissue was cleaned using a forceps and warm PBS and aliquots of whole adipose tissue were snap-frozen in liquid nitrogen. Some adipose tissue was used fresh for immediate measurement of adipocyte size.

*Adipocyte isolation and measurement adipocyte size and number* - Adipose tissue was placed in 0.65 mg/mL collagenase (Collagenase, Sigma-Aldrich, St. Louis, MO, USA) resolved in Media 199 (M199, Gibco, Thermo Fisher Scientific, Waltham, MA, USA) with 4% bovine albumin added at 37°C. The tissue was incubated for 60 min at a gentle agitation at 140 rpm, isolated cells were sieved through a silicone mesh to remove tissue remnants and adipocytes were washed in media 4 times. Adipocytes were suspended in M199 with 4% albumin at approximately 20% (volume/volume density) and added to a hydrophobic object glass coated with silicon oil (5). Cell size was measured using a Zeiss Axiophot microscope (Carl Zeiss AB, Oberkochen, Germany) and an objective with integrated μm scale (Carl Zeiss 4444036-9000). Adipocyte diameter was measured for 100 cells per participant. Adipocyte volume was estimated using the equation $V=\frac{4\pi}{3}\times r^{3}$where V is the volume and r the radius of the adipocyte. The density of adipose tissue has earlier been determined to range between 0.925-0.97 g/ml and thus, the volume of 100 g fat can be approximated. We estimated the mean adipose density to 9.5 g/ml and given the number of adipocytes and the volume of 100 g adipose tissue, the number of adipocytes per 100 g was estimated.

**Laboratory procedures**

*Plasma glucose, plasma FFA and blood tests* - Other analyses performed at the accredited laboratory at Department of Clinical Chemistry, Sahlgrenska University Hospital were measurements of blood samples from the screening visit including haemoglobin, white blood cell count, thrombocyte count, erythrocyte volume fraction, sodium, potassium, calcium, creatinine, U-albumin/creatinine ratio, aspartate aminotransferase, alanine aminotransferase, alkaline phosphatase, bilirubin, triglycerides, total cholesterol, LDL cholesterol, HDL cholesterol, thyroxin, haemoglobin A1c, C-reactive protein, HIV and hepatitis B. The data set have n ≥ 8 for all circulating variables at all-time points in figures and tables representing lean, obese-IR and obese-T2D subjects.

*Interstitial glycerol, lactate and glucose measurements* - Two catheters were always inserted, and the mean value of them was used. One catheter instead of two was used in 2 lean, 1 obese-IR and 1 obese-T2D for assessments of subcutaneous (sc) interstitial glycerol, lactate, and glucose concentrations. I-glycerol and i-lactate showed n ≥ 7 at any time point except at 15 min (n=6) in obese-IR subjects. Similarly, I-glucose showed n ≥ 7 except at 15 min (n=4), at 150 min (n=6) and at 180 min (n=6) in obese-IR subjects.

*Interstitial insulin measurement* - Mean of measurements of 2 microdialysis catheters were used for all participants and we had at least one functional catheter in every subject studied. Lean subjects displayed a considerable number of missing data on interstitial (i) insulin after the OGTT: results at 15 min (n=2), 60 min (n=6), 75 min (n=6), 150 min (n=5) and 180 min (n=5). Further, obese-IR subjects showed missing data on interstitial (i) insulin at 180 min (n=4) and obese-T2D at 15 min (n=6) and at 30 min (n=6). All groups showed i-insulin n ≥ 7 for all the other time points. I-insulin before OGTT displayed great variation because of dialysate values close to the detection limit of the immunoassay.

**Safety data**

No subject displayed increased ear temperature (temp > 37.5°C) at any visit during the MD-Lipolysis Study. One obese-IR subject reported an upper airway infection during the week prior to visit 2 but he felt much better one day before the investigation. The subject had no fever in the morning at visit 2 and we decided to perform the investigation according to plan. After the investigation we obtained a serum CRP at 68 mg/l which is an elevated value. As CRP has a half-life of 19 hrs and the participant had returned to his habitual state with normal body temperature, there were no grounds for exclusion. Another obese-IR exhibited an abnormal lab value but was included in the study as this was not against study criteria for the non-T2D groups. The subject had known proteinuria (16 g/l) because of urostomia secondary to an intervention against a malformation. The creatinine was in the normal range and estimated glomerular filtration rate was > 60 ml/min/1.73 m^2^.

All subjects exhibited a blood pressure after fasting overnight that was 180/110 mm Hg. At check-up before visit 2 and visit 3 a few subjects showed blood pressure ≥ 180/105 mm Hg and because of that we added a calcium blocker, as described.

There was less than 2-fold increase of ALT in a few subjects most likely due to liver steatosis. No other clinically significant deviations of safety lab results were observed.

**Restrictions during study**

The subjects had to be stable in weight (weight changes not more than ± 5 kg) and keep the same lifestyle during the study, be fasting overnight (10 h) before all the visits to the clinic, refrain from alcohol and strenuous exercise (defined as greater than 70% of the maximal pulse rate for 1 h or more) within 48 h prior to all clinic visits (including baseline).

**References**

1. Alberti KG, Zimmet PZ. Definition, diagnosis and classification of diabetes mellitus and its complications. Part 1: diagnosis and classification of diabetes mellitus provisional report of a WHO consultation. Diabet Med 1998;15(7):539-53.

2. Kuk JL, Ardern CI. Are metabolically normal but obese individuals at lower risk for all-cause mortality? Diabetes Care 2009;32(12):2297-9.

3. Sandqvist M, Johanson EH, Ahren B, Axelsen M, Schmelz M, Smith U, et al. Postprandial interstitial insulin concentrations in type 2 diabetes relatives. Eur J Clin Invest 2006;36(6):383-8.

4. Sjostrand M, Holmang A, Lonnroth P. Measurement of interstitial insulin in human muscle. Am J Physiol 1999;276(1):E151-4.

5. Jansson PA, Larsson A, Smith U, Lonnroth P. Glycerol production in subcutaneous adipose tissue in lean and obese humans. J Clin Invest 1992;89(5):1610-7.

**Supplemental figures and figure legends**


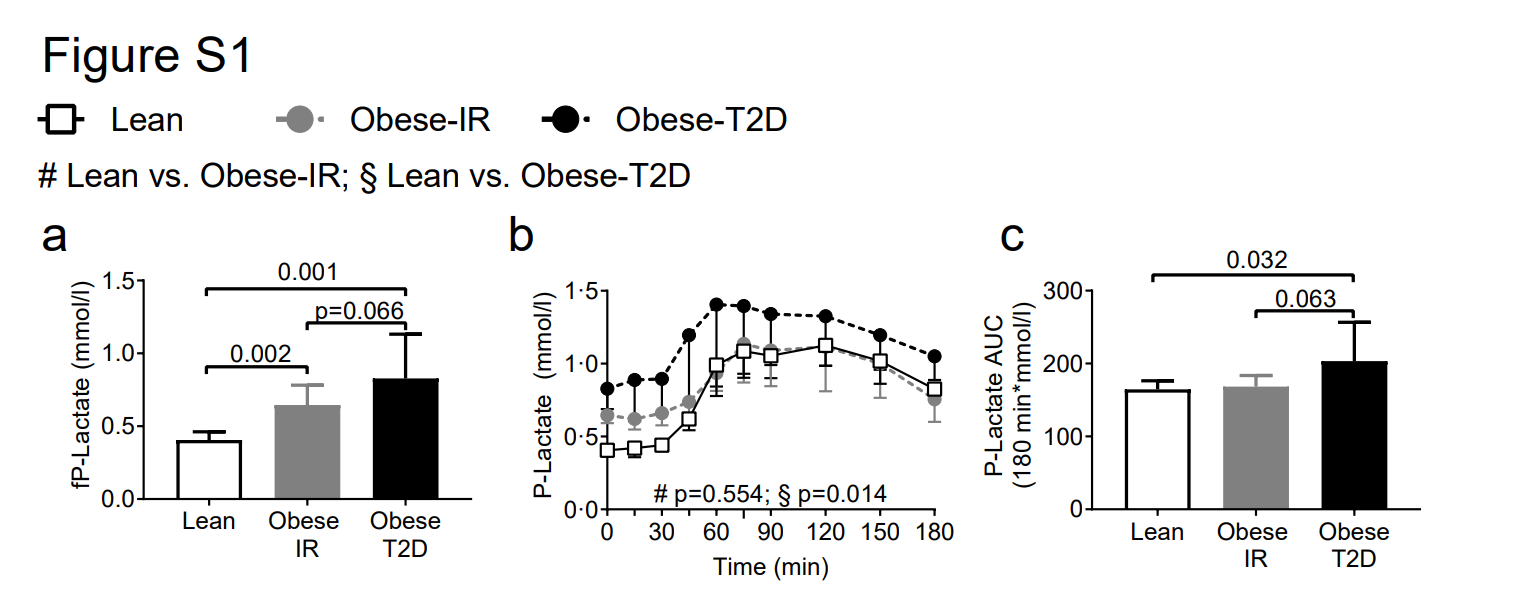
**Figure S1. Fasting plasma lactate of Lean, Obese-IR, and Obese-T2D subjects during OGTT.**

(a) Fasting plasma lactate (fP-Lactate); (b) plasma lactate (P-Lactate), and (c) AUC of b. Data are presented as median and error bars indicate IQR. n=9 for all groups in all panels [Mann-Whitney U-test for bars, mixed-effects models for curves].


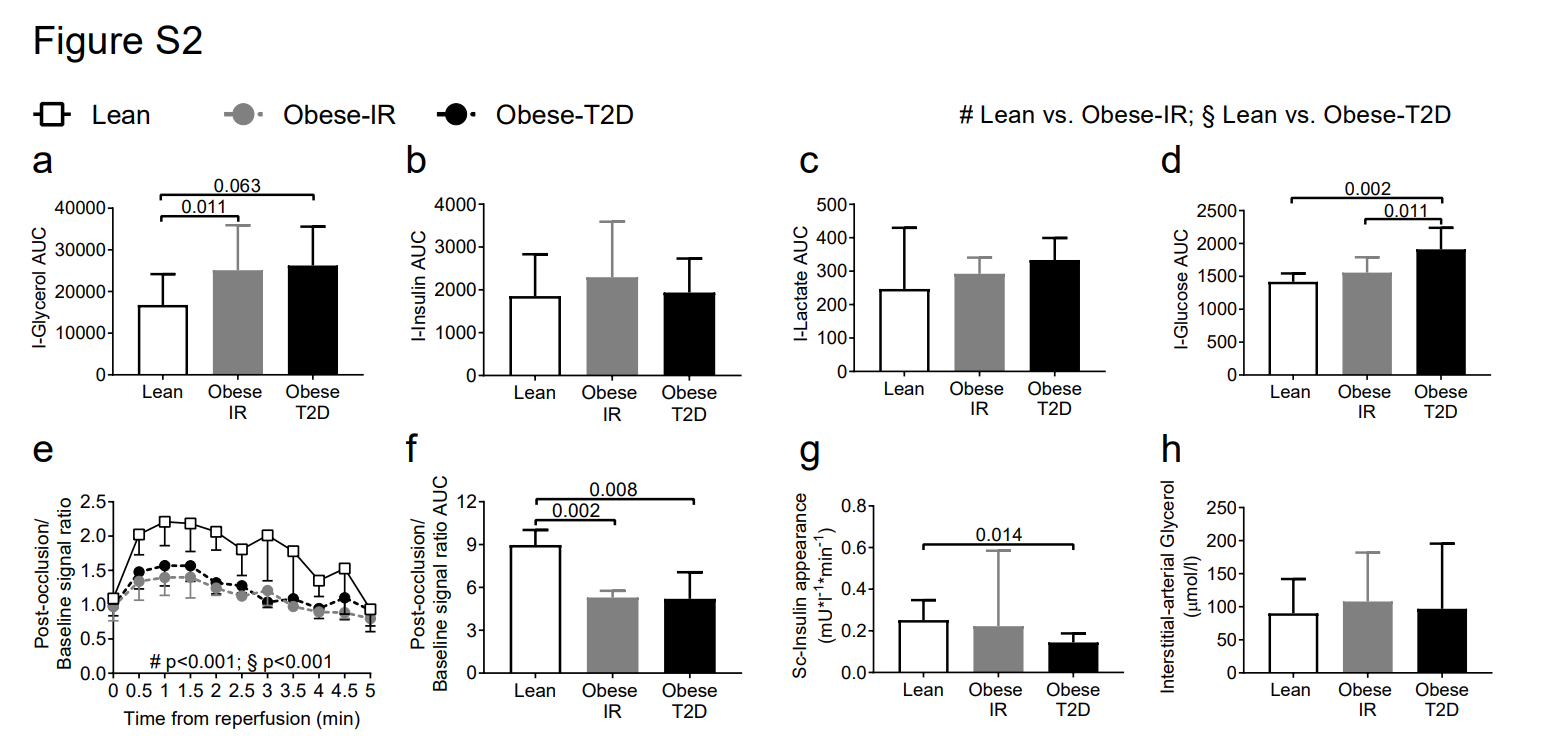
**Figure S2. Interstitial concentrations of metabolites, peripheral microvascular function and insulin appearance rate in abdominal subcutaneous adipose tissue of Lean, Obese-IR, and Obese-T2D subjects.**

(a-d) Areas under the curves, described in Figure 3 a, b, c, d of abdominal subcutaneous interstitial metabolite concentrations measured during the oral glucose tolerance test: (a) AUC for I-glycerol; (b) AUC for I-insulin; (c) AUC for I-lactate; (d) AUC for I-glucose 180 min. (e, f) Peripheral endothelial function measured with the EndoPAT (peripheral arterial tone) device at Visit-3 (e) and 5-min area under the curve (f). (g) Adipose tissue appearance rate of insulin during the OGTT (Visit-2) over the first hour of the experiment. (h) Differences between interstitial and arterialized venous plasma fasting glycerol concentrations in abdominal subcutaneous adipose tissue. Data are presented as median and error bars indicate IQR. n=9 for lean and obese-T2D and n=8 for obese-IR in all panels except (b) where n=9 for all groups, and (e-f) where n=8 for the two obese groups [Mann-Whitney U-test for all bars, mixed-effects models for the curve].

Figure S3


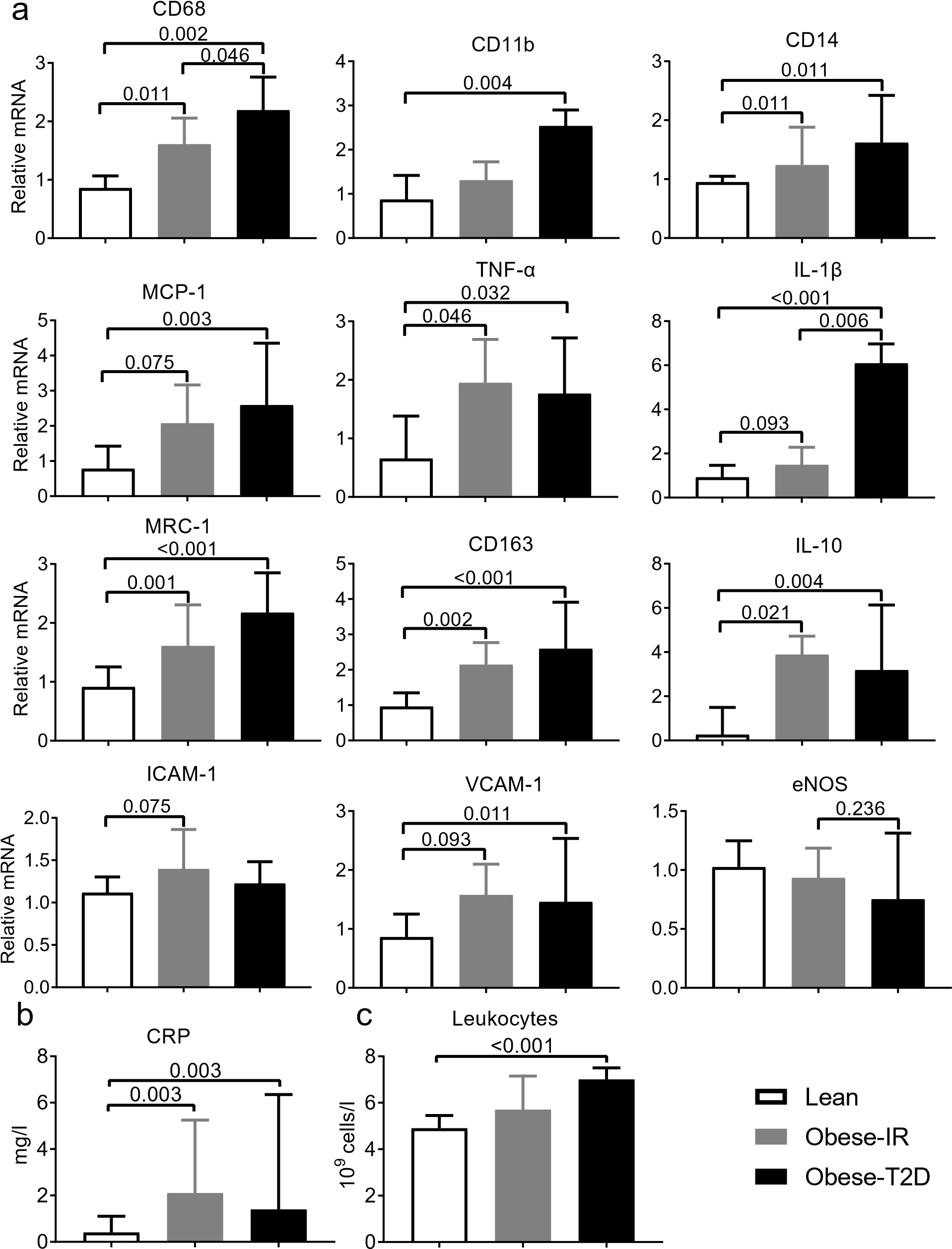


**Figure S3. Adipose tissue metabolic inflammation in the MD-Lipolysis study**

(a) mRNA abundances of markers of adipose tissue inflammation were measured by real-time PCR in abdominal subcutaneous adipose tissue biopsies. (b) Circulating concentrations of C-reactive protein (CRP) and, (c) blood leukocytes counts. Data are presented as median and error bars indicate IQR. a n=9 for Lean and Obese-T2D; and n=8 for Obese-IR. b-c n=9 in all groups [Mann-Whitney U-test].


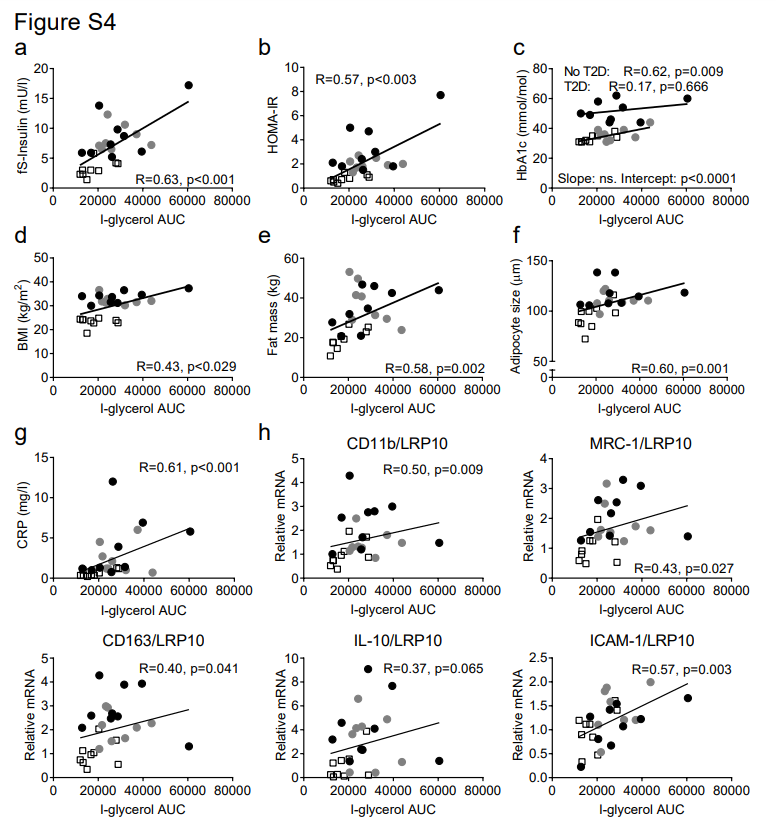


**Figure S4. Correlations of glycerol levels in subcutaneous adipose tissue with measurements of insulin resistance, adiposity, adipocyte size, and inflammation**

Areas under the curves (AUC) of abdominal subcutaneous interstitial glycerol levels (I-Glycerol AUC) during the glucose tolerance test were correlated with different parameters: (a) fasting serum insulin (fS-insulin) vs I-Glycerol AUC; (b) HOMA-IR index vs I-Glycerol AUC; (c) HbA1c vs I-Glycerol AUC; (d) body mass index (BMI) vs I-Glycerol AUC; (e) fat mass vs I-Glycerol AUC; (f) adipocyte size vs I-Glycerol AUC; (g) C-reactive protein (CRP) vs I-Glycerol AUC; (h) adipose tissue mRNA levels of markers of inflammation were correlated with I-glycerol AUC. n=9 for Lean and Obese-T2D, and n=8 for Obese-IR for all panels. p-values: (a, b and d-h) [Spearman´s rank correlation] and (c) [Spearman´s rank correlation, linear regression]

**
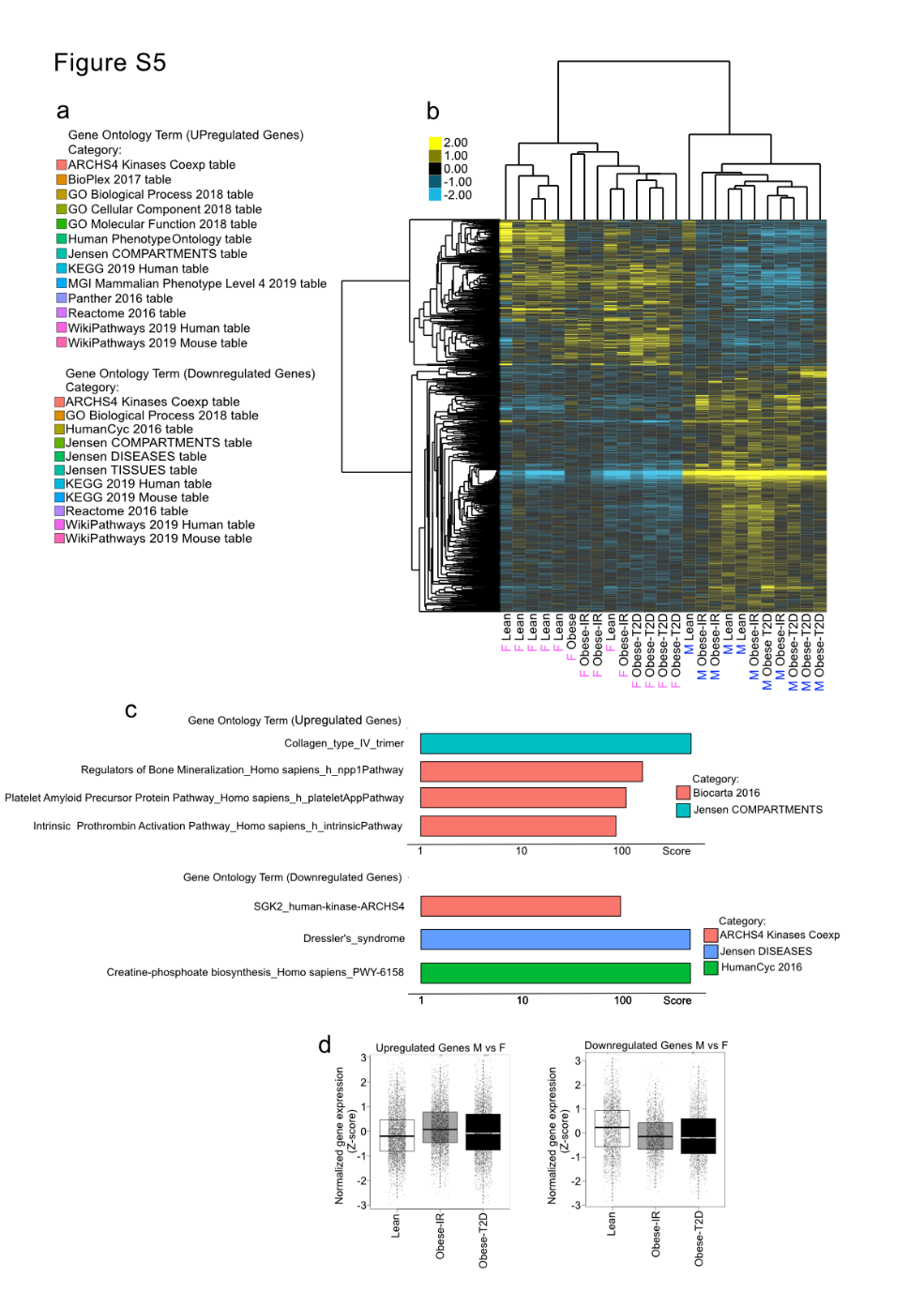
**

**Figure S5. Influence of sex and metabolic subgroup on results from the adipose tissue RNA sequencing analysis in the MD-Lipolysis study.**

(a) Colour code indicating the gene set database in the functional enrichment of Gene Ontology Terms indicated in Figure 4d. (b) Heat maps of males M vs females F differentially expressed genes, normalization and statistical analysis was as in Figure 4a. (c) Bar plots represent the enrichment of the biological terms based on the Combined score (x-axis), as calculated by the EnrichR web tool. All terms have multiple tests correction adjusted p-value less than 0.05. (d) Box plots comparison of the Lean, Obese-IR, and Obese-T2D categories of Male vs. Female differentially expressed genes showing virtually no changes in respect to the patient conditions.

| **Table S1. Medical history and baseline medications of subjects in the MD-Lipolysis study** | | | |
| --- | --- | --- | --- |
|  | **Lean** | **Obese-IR** | **Obese-T2D** |
| **Medical History** |  |  |  |
| **Allergy** |  |  |  |
| Allergy | 3/9 | 0/9 | 2/9 |
| **Cardiovascular/Metabolic disease** |  |  |  |
| Hypertension | 0/9 | 0/9 | 6/9 |
| Hyperlipidemia | 1/9 | 1/9 | 2/9 |
| Fatty liver disease | 0/9 | 0/9 | 1/9 |
| **Endocirine disease** |  |  |  |
| Hypothyroidism | 0/9 | 1/9 | 1/9 |
| **Eye disease** |  |  |  |
| Catarract | 0/9 | 1/9 | 0/9 |
| Glaucoma | 0/9 | 1/9 | 0/9 |
| **Gastrointestinal disorder** |  |  |  |
| Gastroesophageal reflux | 0/9 | 1/9 | 1/9 |
| IBS | 0/9 | 1/9 | 1/9 |
| Diverticulitis | 1/9 | 0/9 | 0/9 |
| **Infection/inflammation** |  |  |  |
| Prostatitis | 1/9 | 0/9 | 0/9 |
| Sepsis | 0/9 | 0/9 | 1/9 |
| Mononucleosis | 1/9 | 0/9 | 0/9 |
| Psoriasis arthritis | 0/9 | 0/9 | 1/9 |
| **Musculoskeletal disorder** |  |  |  |
| Lumbar pain | 0/9 | 0/9 | 1/9 |
| osteopenia | 0/9 | 1/9 | 0/9 |
| Whiplash | 1/9 | 0/9 | 0/9 |
| Rib fracture | 1/9 | 0/9 | 0/9 |
| Spinal stenosis | 0/9 | 1/9 | 0/9 |
| Arthralgy | 1/9 | 0/9 | 0/9 |
| Spina bifida | 0/9 | 1/9 | 0/9 |
| **Nervous system/psychiatric disorder** |  |  |  |
| Depression | 0/9 | 1/9 | 1/9 |
| Stress | 1/9 | 0/9 | 0/9 |
| Sleeping disorder | 0/9 | 1/9 | 0/9 |
| Migraine | 1/9 | 0/9 | 0/9 |
| **Orthopedic disease** |  |  |  |
| Arthroscopy knee | 2/9 | 0/9 | 0/9 |
| Clavicle fracture | 2/9 | 0/9 | 0/9 |
| Ganglion op | 2/9 | 0/9 | 0/9 |
| Hip fracture (traumatic) | 1/9 | 0/9 | 0/9 |
| Monoartrhitis | 1/9 | 0/9 | 0/9 |
| Hip infection | 1/9 | 0/9 | 0/9 |
| Hand fracture | 1/9 | 0/9 | 0/9 |
| Disc prolaps surgery | 1/9 | 0/9 | 0/9 |
| Achilles tendon surgery | 1/9 | 0/9 | 0/9 |
| Carpal tunnel syndrome | 0/9 | 1/9 | 0/9 |
| Osteoarthritis | 0/9 | 1/9 | 0/9 |
| Tibia fracture | 0/9 | 0/9 | 1/9 |
| **Other disease** |  |  |  |
| B12 deficiency | 0/9 | 0/9 | 2/9 |
| Endometriosis | 0/9 | 1/9 | 0/9 |
| Varicose veins | 0/9 | 1/9 | 0/9 |
| Cervical dysplasia | 0/9 | 0/9 | 1/9 |
| **Respiratory disease** |  |  |  |
| Asthma | 0/9 | 1/9 | 1/9 |
| Pulmonary embolus | 0/9 | 1/9 | 1/9 |
| Sleep apnea | 0/9 | 0/9 | 2/9 |
| Pneumothorax | 1/9 | 0/9 | 0/9 |
| **Surgery/intervention** |  |  |  |
| Appendicitis | 1/9 | 2/9 | 2/9 |
| Tonsillectomy | 2/9 | 0/9 | 0/9 |
| Caesarean section | 1/9 | 0/9 | 1/9 |
| Hysterectomy | 0/9 | 0/9 | 1/9 |
| Retinal amotio | 0/9 | 1/9 | 0/9 |
| Gall disease | 1/9 | 0/9 | 1/9 |
| Infection surgery | 0/9 | 1/9 | 1/9 |
| Umbilical prolaps | 0/9 | 0/9 | 1/9 |
| Lipoma | 0/9 | 1/9 | 0/9 |
| **Urinary tract disorder** |  |  |  |
| Benign prostate hyperplasia | 1/9 | 1/9 | 0/9 |
| Urinary tract infection | 1/9 | 0/9 | 0/9 |
| Overactive bladder | 0/9 | 0/9 | 1/9 |
| Urostomia | 0/9 | 1/9 | 0/9 |
|  |  |  |  |
| **Baseline Medication** |  |  |  |
| **Alfa-1-receptor blocker** |  |  |  |
| Alfuzosin | 0/9 | 1/9 | 0/9 |
| **Analgetics** |  |  |  |
| Paracetamol PRN | 3/9 | 0/9 | 0/9 |
| **Anti-depressants** |  |  |  |
| Fluoxetine | 0/9 | 1/9 | 0/9 |
| Citalopram | 0/9 | 0/9 | 1/9 |
| **Anti-diabetic medication** |  |  |  |
| Metformin | 0/9 | 0/9 | 8/9 |
| **Anti-histamines** |  |  |  |
| Ebastin | 1/9 | 0/9 | 1/9 |
| Sodium cromoglycate | 1/9 | 0/9 | 0/9 |
| **Anti-hypertensive medication** |  |  |  |
| Amlodipine | 0/9 | 0/9 | 3/9 |
| Felodipine | 0/9 | 0/9 | 2/9 |
| Enalapril | 0/9 | 0/9 | 1/9 |
| Lisinopril | 0/9 | 0/9 | 1/9 |
| Candesartan | 0/9 | 0/9 | 1/9 |
| **Beta-receptor agonist** |  |  |  |
| Salbutamol PRN | 0/9 | 0/9 | 1/9 |
| **Glucocorticoids** |  |  |  |
| Flutikasonpropionate (nasal) | 0/9 | 0/9 | 1/9 |
| **Hormones and hormone modulators** |  |  |  |
| Finasteride | 0/9 | 1/9 | 0/9 |
| Levaxin | 0/9 | 1/9 | 1/9 |
| Estrogen patch | 0/9 | 0/9 | 1/9 |
| **Hypnotics** |  |  |  |
| Propiomazine | 0/9 | 1/9 | 0/9 |
| **Lipid lowering drugs** |  |  |  |
| Simvastatin | 0/9 | 0/9 | 2/9 |
| Atorvastatin | 1/9 | 1/9 | 0/9 |
| **Non-steroid anti-inflammatory drugs** |  |  |  |
| Ibuprofen PRN | 3/9 | 0/9 | 0/9 |
| Diclofenac | 2/9 | 0/9 | 0/9 |
| Etoricoxib | 0/9 | 1/9 | 0/9 |
| **Other medications** |  |  |  |
| Montelukast | 0/9 | 0/9 | 1/9 |
| Fesoterodinfumarate | 0/9 | 0/9 | 1/9 |
| Dimetikon | 0/9 | 1/9 | 0/9 |
| **Proton-pump inhibitors** |  |  |  |
| Omeprazol | 0/9 | 2/9 | 0/9 |
| **Supplements** |  |  |  |
| Calcium | 0/9 | 1/9 | 0/9 |
| **Triptans** |  |  |  |
| Imigrane | 1/9 | 0/9 | 0/9 |
| **Urinary antiseptics** |  |  |  |
| Methenamine hippurate | 0/9 | 1/9 | 0/9 |
| **Vitamins** |  |  |  |
| Vitamin B12 | 0/9 | 0/9 | 2/9 |
| Vitamin D | 2/9 | 1/9 | 0/9 |
| Data are presented as n/group n. |  |  |  |

| **Table S2. Reasons for exclusion of participants at screening and protocol violations** | | |
| --- | --- | --- |
| **in the MD-Lipolysis study** | | |
| **Group** | **Sex** | **Exclusion** |
| Lean | ♂ | BMI > 25 kg/m^2^ |
| Lean | ♂ | BMI > 25 kg/m^2^ |
| Lean | ♀ | Declined participation |
| Lean | ♀ | Back pain |
| Lean | ♂ | High nicotine consumption |
| Lean | ♀ | Failed the oral glucose tolerance test |
| Lean | ♂ | Vasovagal reaction at screening |
| Lean | ♀ | Vigorous exercise day before Visit-2* |
| Lean | ♂ | Alcohol consumption day before Visit-2* |
| Lean | ♂ | Pronounced vasovagal reaction on Visit-2* |
| Lean | ♀ | Inaccessible veins |
| Obese-IR | ♂ | Declined participation |
| Obese-IR | ♀ | Declined participation |
| Obese-IR | ♀ | Declined participation |
| Obese-IR | ♀ | Declined participation |
| Obese-IR | ♂ | Declined participation |
| Obese-IR | ♀ | Low fasting insulin |
| Obese-IR | ♀ | Low fasting insulin |
| Obese-IR | ♀ | Low fasting insulin |
| Obese-IR | ♀ | Low fasting insulin |
| Obese-IR | ♀ | Low fasting insulin |
| Obese-IR | ♂ | Low fasting insulin |
| Obese-IR | ♂ | BMI < 30 kg/m^2^ |
| Obese-IR | ♀ | BMI < 30 kg/m^2^ |
| Obese-IR | ♀ | Inaccessible veins |
| Obese-IR | ♀ | Previous venous thrombosis |
| Obese-IR | ♀ | Significant albuminuria discovered at Visit-1 |
| Obese-IR | ♀ | Alcohol misuse |
| Obese-IR | ♂ | Rapid weight loss |
| Obese-T2D | ♂ | Rapid weight loss |
| Obese-T2D | ♀ | Rapid weight loss |
| Obese-T2D | ♂ | Poor metabolic control |
| Obese-T2D | ♂ | Poor metabolic control |
| Obese-T2D | ♀ | Back pain |
| Obese-T2D | ♂ | Back pain |
| Obese-T2D | ♂ | BMI < 30 kg/m^2^ |
| Obese-T2D | ♂ | Inaccessible veins |
| Obese-T2D | ♂ | Testosterone misuse |
| Obese-T2D | ♀ | > 6 years duration of type 2 diabetes |
| Obese-T2D | ♀ | Initiated a caloric restricted diet between Visit-1 and Visit-2* |
| BMI: body mass index | | |
| *Excluded due to protocol violation. | | |

| **Table S3. Comparisons of metabolic and vascular variables between Lean, Obese-IR and Obese-T2D subjects during OGTT in the MD-Lipolysis study** | | | | | | | | | |
| --- | --- | --- | --- | --- | --- | --- | --- | --- | --- |
|  |  | **Lean vs. Obese-IR** | |  | **Lean vs. Obese-T2D** | |  | **Obese-IR vs. Obese-T2D** | |
|  |  | **Mean difference (CI)** | **p-value** |  | **Mean difference (CI)** | **p-value** |  | **Mean difference (CI)** | **p-value** |
| P-Glucose (mmol/l) |  | -0.6 (-1.8; 0,6) | 0.329 |  | -5.7 (-7.5; -3.9) | <0.001 |  | -5.1 (-7.1; -3.1) | <0.001 |
| S-Insulin (mU/l) |  | -18.0 (-36.9; 1.0) | 0.062 |  | -1.8 (-10.1; 6.5) | 0.646 |  | 16.1 (-2.5; 34.8) | 0.085 |
| P-Glycerol (µmol/l) |  | -26 (-46; -5) | 0.017 |  | -23 (-42; -6) | 0.013 |  | 2 (-24; 28) | 0.872 |
| P-FFA (mmol/l) |  | -0.15 (-0.22; -0.10) | 0.002 |  | -0.22 (-0.27; -0.16) | <0.001 |  | -0.06 (-0.14; 0.02) | 0.141 |
| P-Lactate (mmol/l) |  | 0.0 (-0.2; 0.1) | 0.554 |  | -0.3 (-0.5; -0.1) | 0.014 |  | -0.2 (-0.5; 0.0) | 0.059 |
| I-Glycerol (µmol/l) |  | -61 (-102; -19) | 0.008 |  | -61 (-123; 1) | 0.053 |  | -1 (-70; 68) | 0.981 |
| I-Insulin (mU/l) |  | -8.5 (-23.3; 6.3) | 0.242 |  | 1.4 (-3.7; 6.4) | 0.577 |  | 9.7 (-4.5; 24.0) | 0.167 |
| I-Lactate (mmol/l) |  | 0.1 (-0.5; 0.6) | 0.821 |  | -0.2 (-0.8; 0.3) | 0.400 |  | -0.3 (-0.6; 0.1) | 0.112 |
| I-Glucose (mmol/l) |  | -1.5 (-3.3; 0.3) | 0.100 |  | -3.5 (-5.1; -1.8) | 0.001 |  | -1.9 (-3.7; -0.1) | 0.040 |
| ATBF (ml/[100g*min)]) |  | 2.1 (1.0; 3.2) | <0.001 |  | 2.0 (0.9; 3.1) | <0.001 |  | -0.1 (-0.7; 0.5) | 0.652 |
| EndoPAT |  | 0.6 (0.3; 0.9) | <0.001 |  | 0.6 (0.3; 0.9) | <0.001 |  | 0.0 (-0.3; 0.3) | 0.924 |
| CI: 95% confidence interval; FFA: free fatty acids; ATBF: adipose tissue blood flow; EndoPAT: Post-occlusion/Baseline signal ratio of the EndoPAT measurement. | | | | | | | | | |
| Difference in predicted means and p-values from mixed-effects models of time dependant metabolic variables in MD-lipolysis study during the OGTT.  P-value < 0.05 is considered statistically significant. | | | | | | | | | |
