# Supplementary material for: Hyperinsulinemia and insulin resistance in the obese may develop as part of a homeostatic response to elevated free fatty acids: A mechanistic case-control and a population-based cohort study
Source: eBioMedicine. 2021 Mar 9;65:103264. doi: 10.1016/j.ebiom.2021.103264 (PMC7992078; doi:10.1016/j.ebiom.2021.103264)
Supplement: Supplementary file 2 [file mmc2.docx]

STROBE Statement—Checklist of items that should be included in reports of the MD-Lipolysis, ***case-control study***

|  | Item No | Recommendation |
| --- | --- | --- |
| **Title and abstract** | 1 | (*a*) Indicate the study’s design with a commonly used term in the title or the abstract  -Page 1-2 |
|  |  | (*b*) Provide in the abstract an informative and balanced summary of what was done and what was found  -Page 2 |
| Introduction | | |
| Background/rationale | 2 | Explain the scientific background and rationale for the investigation being reported  -Page 4 |
| Objectives | 3 | State specific objectives, including any prespecified hypotheses  -Page 6 |
| Methods | | |
| Study design | 4 | Present key elements of study design early in the paper  -Page 5-6 |
| Setting | 5 | Describe the setting, locations, and relevant dates, including periods of recruitment, exposure, follow-up, and data collection  -Page 6 |
| Participants | 6 | (*a*) Give the eligibility criteria, and the sources and methods of case ascertainment and control selection. Give the rationale for the choice of cases and controls  -Page 6-7, Figure 1a |
|  |  | (*b*) For matched studies, give matching criteria and the number of controls per case  -Figure 1a |
| Variables | 7 | Clearly define all outcomes, exposures, predictors, potential confounders, and effect modifiers. Give diagnostic criteria, if applicable  -Page 7-11 |
| Data sources/ measurement | 8* | For each variable of interest, give sources of data and details of methods of assessment (measurement). Describe comparability of assessment methods if there is more than one group  -Page 7-11 |
| Bias | 9 | Describe any efforts to address potential sources of bias  -Page 7 |
| Study size | 10 | Explain how the study size was arrived at  -Page 6 |
| Quantitative variables | 11 | Explain how quantitative variables were handled in the analyses. If applicable, describe which groupings were chosen and why  -Page 13 |
| Statistical methods | 12 | (*a*) Describe all statistical methods, including those used to control for confounding  -Page 13 |
|  |  | (*b*) Describe any methods used to examine subgroups and interactions  -Page 12 |
|  |  | (*c*) Explain how missing data were addressed  -Supplementary methods |
|  |  | (*d*) If applicable, explain how matching of cases and controls was addressed  -Figure 1a |
|  |  | (*e*) Describe any sensitivity analyses  -No specific sensitivity analysis is detailed. However, key findings from MD-Lipolysis study are verified in an independent cohort (POEM). |
| Results | | |
| Participants | 13* | (a) Report numbers of individuals at each stage of study—eg numbers potentially eligible, examined for eligibility, confirmed eligible, included in the study, completing follow-up, and analysed  -Figure 1a |
|  |  | (b) Give reasons for non-participation at each stage  -Figure 1a, Table S2 |
|  |  | (c) Consider use of a flow diagram  -Figure 1a |
| Descriptive data | 14* | (a) Give characteristics of study participants (eg demographic, clinical, social) and information on exposures and potential confounders  -Table 1 |
|  |  | (b) Indicate number of participants with missing data for each variable of interest  -Supplementary methods. Figure legends. |
| Outcome data | 15* | Report numbers in each exposure category, or summary measures of exposure  -Figure 1a, Table 1 |
| Main results | 16 | (*a*) Give unadjusted estimates and, if applicable, confounder-adjusted estimates and their precision (eg, 95% confidence interval). Make clear which confounders were adjusted for and why they were included  -Table 1, Figure 1-3, Figure S1-3. |
|  |  | (*b*) Report category boundaries when continuous variables were categorized  -No categorized continous variables were used. |
|  |  | (*c*) If relevant, consider translating estimates of relative risk into absolute risk for a meaningful time period  -No relative risks are presented. |

| Other analyses | 17 | Report other analyses done—eg analyses of subgroups and interactions, and sensitivity analyses  -No subgroup analyses were done. |
| --- | --- | --- |
|  |  |  |
| Discussion | | |
| Key results | 18 | Summarise key results with reference to study objectives  -Page 24-25 |
| Limitations | 19 | Discuss limitations of the study, taking into account sources of potential bias or imprecision. Discuss both direction and magnitude of any potential bias  -Page 27 |
| Interpretation | 20 | Give a cautious overall interpretation of results considering objectives, limitations, multiplicity of analyses, results from similar studies, and other relevant evidence  -Page 24-27 |
| Generalisability | 21 | Discuss the generalisability (external validity) of the study results  -Page 27-28 |
| Other information | | |
| Funding | 22 | Give the source of funding and the role of the funders for the present study and, if applicable, for the original study on which the present article is based  -Page 3 |

*Give information separately for cases and controls.

**Note:** An Explanation and Elaboration article discusses each checklist item and gives methodological background and published examples of transparent reporting. The STROBE checklist is best used in conjunction with this article (freely available on the Web sites of PLoS Medicine at http://www.plosmedicine.org/, Annals of Internal Medicine at http://www.annals.org/, and Epidemiology at http://www.epidem.com/). Information on the STROBE Initiative is available at http://www.strobe-statement.org.
