# Supplementary material for: Hyperinsulinemia and insulin resistance in the obese may develop as part of a homeostatic response to elevated free fatty acids: A mechanistic case-control and a population-based cohort study
Source: eBioMedicine. 2021 Mar 9;65:103264. doi: 10.1016/j.ebiom.2021.103264 (PMC7992078; doi:10.1016/j.ebiom.2021.103264)
Supplement: Supplementary file 3 [file mmc3.docx]

STROBE Statement—Checklist of items that should be included in reports of the POEM ***cohort study***

|  | Item No | Recommendation |
| --- | --- | --- |
| **Title and abstract** | 1 | (*a*) Indicate the study’s design with a commonly used term in the title or the abstract -Page 2 |
|  |  | (*b*) Provide in the abstract an informative and balanced summary of what was done and what was found  -Page 2 |
| Introduction | | |
| Background/rationale | 2 | Explain the scientific background and rationale for the investigation being reported  -Page 4 |
| Objectives | 3 | State specific objectives, including any prespecified hypotheses  -Page 6 |
| Methods | | |
| Study design | 4 | Present key elements of study design early in the paper  -Page 5-6 |
| Setting | 5 | Describe the setting, locations, and relevant dates, including periods of recruitment, exposure, follow-up, and data collection  -Page 11 |
| Participants | 6 | (*a*) Give the eligibility criteria, and the sources and methods of selection of participants. Describe methods of follow-up  -Page 11 |
|  |  | (*b*) For matched studies, give matching criteria and number of exposed and unexposed  -Not a matched study. |
| Variables | 7 | Clearly define all outcomes, exposures, predictors, potential confounders, and effect modifiers. Give diagnostic criteria, if applicable  -Supplementary methods. |
| Data sources/ measurement | 8* | For each variable of interest, give sources of data and details of methods of assessment (measurement). Describe comparability of assessment methods if there is more than one group  -Page 13 |
| Bias | 9 | Describe any efforts to address potential sources of bias  -Page 12 |
| Study size | 10 | Explain how the study size was arrived at  -Page 12 |
| Quantitative variables | 11 | Explain how quantitative variables were handled in the analyses. If applicable, describe which groupings were chosen and why  -Page 13, 21-22 |
| Statistical methods | 12 | (*a*) Describe all statistical methods, including those used to control for confounding  -Page 13 |
|  |  | (*b*) Describe any methods used to examine subgroups and interactions  -Page 13 |
|  |  | (*c*) Explain how missing data were addressed  -Page 12 |
|  |  | (*d*) If applicable, explain how loss to follow-up was addressed  -POEM is a cross-sectional study. |
|  |  | (*e*) Describe any sensitivity analyses  -The POEM cohort was used to validate the MD-Lipolysis study. No sensitivity analysis was done in the POEM cohort study. |
| Results | | |
| Participants | 13* | (a) Report numbers of individuals at each stage of study—eg numbers potentially eligible, examined for eligibility, confirmed eligible, included in the study, completing follow-up, and analysed  -Figure 5a |
|  |  | (b) Give reasons for non-participation at each stage  -Figure 5a |
|  |  | (c) Consider use of a flow diagram  -Figure 5a |
| Descriptive data | 14* | (a) Give characteristics of study participants (eg demographic, clinical, social) and information on exposures and potential confounders  -Table 2, Page 12. |
|  |  | (b) Indicate number of participants with missing data for each variable of interest  -No missing data. |
|  |  | (c) Summarise follow-up time (eg, average and total amount)  -No follow up. |
| Outcome data | 15* | Report numbers of outcome events or summary measures over time |
| Main results | 16 | (*a*) Give unadjusted estimates and, if applicable, confounder-adjusted estimates and their precision (eg, 95% confidence interval). Make clear which confounders were adjusted for and why they were included  -Page 5b-g |
|  |  | (*b*) Report category boundaries when continuous variables were categorized  -No categorized variables. |
|  |  | (*c*) If relevant, consider translating estimates of relative risk into absolute risk for a meaningful time period  -No relative risk. |
| Other analyses | 17 | Report other analyses done—eg analyses of subgroups and interactions, and sensitivity analyses  -Page 22-23 |
| Discussion | | |
| Key results | 18 | Summarise key results with reference to study objectives  -Page 27 |
| Limitations | 19 | Discuss limitations of the study, taking into account sources of potential bias or imprecision. Discuss both direction and magnitude of any potential bias  -Page 27 |
| Interpretation | 20 | Give a cautious overall interpretation of results considering objectives, limitations, multiplicity of analyses, results from similar studies, and other relevant evidence  -Page 28 |
| Generalisability | 21 | Discuss the generalisability (external validity) of the study results  -Page 27-28 |
| Other information | | |
| Funding | 22 | Give the source of funding and the role of the funders for the present study and, if applicable, for the original study on which the present article is based  -Page 29 |

*Give information separately for exposed and unexposed groups.

**Note:** An Explanation and Elaboration article discusses each checklist item and gives methodological background and published examples of transparent reporting. The STROBE checklist is best used in conjunction with this article (freely available on the Web sites of PLoS Medicine at http://www.plosmedicine.org/, Annals of Internal Medicine at http://www.annals.org/, and Epidemiology at http://www.epidem.com/). Information on the STROBE Initiative is available at http://www.strobe-statement.org.
