# Supplementary material for: Hyperinsulinemia and insulin resistance in the obese may develop as part of a homeostatic response to elevated free fatty acids: A mechanistic case-control and a population-based cohort study
Source: eBioMedicine. 2021 Mar 9;65:103264. doi: 10.1016/j.ebiom.2021.103264 (PMC7992078; doi:10.1016/j.ebiom.2021.103264)
Supplement: Supplementary file 4 [file mmc4.pdf]

UNIVERSITY OF GOTHENBURG

THE INSTITUTE OF MEDICINE

To whom it may concern:

I give permission to be mentioned in writing for editing support in the recent manuscript by Per-Anders Jansson and colleagues, submitted to eBioMedicine for publication.

Sincerely,

Reghan Borer

Institute of Medicine
PO Box 100, SE 405 30 Gothenburg, Sweden
+46 31 342 1000
www.medicine.gu.se
