# Supplementary material for: Hyperinsulinemia and insulin resistance in the obese may develop as part of a homeostatic response to elevated free fatty acids: A mechanistic case-control and a population-based cohort study
Source: eBioMedicine. 2021 Mar 9;65:103264. doi: 10.1016/j.ebiom.2021.103264 (PMC7992078; doi:10.1016/j.ebiom.2021.103264)
Supplement: Supplementary file 5 [file mmc5.docx]

**Pathophysiology associated with overweight and type 2 diabetes**

**Study design:** Study is a case-control study comparing differences in vascular function and the fibrinolysis inhibitor PAI-1 between individuals with type 2 diabetes, individuals with obesity and healthy controls. Research questions the study intend to find answers to is:

**Question 1:** How does vascular function diverge between patients with type 2 diabetes, individuals with obesity, and healthy controls? Which consequences does potential differences have on insulin effects in adipose tissue and muscle tissue?

**Question 2:** What are the differences between patients with type 2 diabetes, individuals with obesity, and healthy controls, when it comes to PAI-1 in blood plasma and thrombocytes at a protein and gene level? How does the aggregation ability differ between the groups?

**Background:** Overweight persons have an increased risk of developing type 2 diabetes and they exhibit a delayed transport of insulin over the blood vessel wall to the target cells in muscle and fat tissue. Normally permeability of insulin increases in the smaller vessels when you for example drink a glucose solution. When this permeability is deficient, we believe that the glucose uptake is affected negatively and as a consequence blood glucose increases. Muscle and fat tissue are target organs for insulin, in other words, in both of these tissues the important effects of the hormone are executed. We are therefore interested in studying the insulin transport over the vessel wall in these tissues in normal weight and overweight persons, and in patients with type 2 diabetes. Differences between these groups in insulin uptake from blood to tissue may clarify metabolic disturbances and why persons develop type 2 diabetes and may also lead to the development of new treatment.

Platelets or thrombocytes are small, without nucleus and circulate in the blood. When a blood vessel is damaged the platelets are activated and contributes to the formation of a blood clot that blocks the damage. During this clot formation tissue plasminogen activator (tPA) is released from the vessel wall, which acts by breaking down the clot to limit its extent. Platelets contains several different proteins, among these plasminogen activator inhibitor-1 (PAI-1) which inhibits the anticoagulant protein tPA. Increased levels of PAI-1 in the blood is a risk factor for myocardial infarction and stroke. High levels of PAI-1 have been measured in persons with obesity and type 2 diabetes. However, the mechanisms underlying elevated PAI-1 levels are unknown. We need to learn more about why PAI-1 levels in blood are increased in type 2 diabetes and why this patient group have an increased risk of developing cardiovascular disease.

**Study plan:** 15 persons with type 2 diabetes (BMI: 30-40) will be included in the study, and in addition will 15 gender, age, and BMI matched controls be recruited. The participants will come in for a screening visit 10-14 days prior to the study, where the general health is examined, blood samples are taken, and instructions of how the participant will stop their medication before visit 2 and 3 are explained. If needed a control of blood pressure and blood glucose are performed 3-5 days prior to visit 2. During visit 2, venous blood sampling, oral glucose tolerance test (OGTT), subcutaneous blood flow measurement and microdialysis will be performed. After the visit will the patient begin to take their medications. A final visit with a subcutaneous (sc) adipose tissue biopsy occurs around 2 weeks after visit 2.

**Implementation:** The study is expected to start during spring 2013 and continue during one year.

**Recruitment of research participants:** Through advertisement or referral from outpatient clinics.

**Inclusion criteria:** Men and women with type 2 diabetes

Age: Men 50-70 years, postmenopausal women 55-70 years (no menstruation during one year and FSH > 40 IU/ml)

BMI: 30-40 kg/m^2^ (type 2 diabetes and obesity); BMI: 18-25 kg/m^2^ (healthy controls)

Healthy obese: fs-insulin > 13 mU/l

Diabetes duration < 5 år

**Exclusion criteria:** Significant cardiovascular disease (except essential hypertension), according to study doctor

Smoking or other significant nicotine addiction, according to study doctor

Significant complication from eyes, kidneys or nerves due to type 2 diabetes, according to study doctor

Treatment with beta-blockers

Treatment with insulin, GLP-1 analogues (Byetta^®^ and Victoza^®^), glitazones and DPP4-inhibitors.

Hematological diseases

Prone to bleeding due to disease or medication, according to study doctor

Other circumstances that complicate participation, e.g. mental illness or addiction.

**Medicines:**

**The following medication shall not be taken during 10 days prior to visit 2 and 3:
Per oral antidiabetics** (SU-compounds, glinides, metformin, acarbose)

**ASA/Statins (**ASA only included if primary prophylaxis)

**Hormones** (Estrogen/Progesterone)

**Anti-hypertensive medication**

**Visit 1:** Occurs 10-14 days prior to visit 2, takes around 2 hours. A general health examination is performed and information on how to take medications is provided. Blood glucose and blood pressure are controlled. Information on diet, exercise and thrombocyte inhibiting medication are specified. See protocol for visit 1.

**Intermediate control:** Occurs 3-5 days before visit 2 for participants where medication is suspended, the visit takes around 20 minutes. Blood glucose and blood pressure are controlled. If mean blood pressure from two measurement are >180/105 mmHg the participant is excluded from the study. See protocol for intermediate control.

**Visit 2:** Study visit 2 takes around 8 hours. The participant arrives at the hospital fasting from 10 pm the evening before. Venous sampling occurs between 8-10 am, thereafter an Oral Glucose Tolerance Test (OGTT, 75g) combined with microdialysis are performed. At the end of the day any medications are reinstated. See protocol for visit 2.

**Visit 3:** A miniature liposuction of subcutaneous adipose tissue is performed, i.e. an adipose tissue needle biopsy. Endothelial function is measured with the EndoPAT method. A clip is attached to the index finger and the blood flow reaction is measured after a blood pressure cuff has been used to apply and release stasis in the upper arm. Fasting plasma glucose and a few blood samples for extra storage of serum and plasma is taken for the biobank. The visit takes around 1.5 hours.

**Pathophysiology associated with overweight and type 2 diabetes**

Personal data for storage:

Allocation number: __________________________________________

Name: __________________________________________

Personal identity number:__________________________________________

Address: __________________________________________

Phone number: __________________________________________

Date: ______________________________________

Investigator:______________________________________

**Pathophysiology associated with overweight and type 2 diabetes**

Visit 1

Allocation number: _________

Date: ____________________________

Investigator:________________________

Age: _________________Gender:___________

Signed informed consent? No [ ] Yes [ ]

(necessary before examination can continue)

Fulfill inclusion criteria? No [ ] Yes [ ]

Medical history (Ask about Viagra^®^, diet supplements and SSRI medication): ___________________________________________________________________________

___________________________________________________________________________

___________________________________________________________________________

___________________________________________________________________________

___________________________________________________________________________

Type 2 diabetes No [ ] Yes [ ]

If yes, medication and doses: ___________________________________________________

___________________________________________________________________________

Hypertension: No [ ] Yes [ ]

If yes, medication and doses: __________________________________________

Other diseases: No [ ] Yes [ ]

If yes, medication and doses: __________________________________________

___________________________________________________________________________

___________________________________________________________________________

Allergy against medication: No [ ] Yes [ ]

If yes, against what and which reaction appeared: ___________________________________

Ex-smoker: No [ ] Yes [ ]

If yes, since when, and how many packages/day:__________________

Exercise (>20 min) [ ] <1 x/week [ ] 1-3 x/week [ ] > 3 x/week [ ]

Status:

Length: _______cm Weight: ________kg Waist: ________cm Hip: _________cm

Sleep, dietary and living habits:

___________________________________________________________________________

___________________________________________________________________________

___________________________________________________________________________

___________________________________________________________________________

___________________________________________________________________________

___________________________________________________________________________

___________________________________________________________________________

Other comments (e.g. easy to identify and puncture veins in the forearm):

___________________________________________________________________________

___________________________________________________________________________

___________________________________________________________________________

___________________________________________________________________________

___________________________________________________________________________

Blood pressure: / mmHg (*lying down left arm, after 10 min rest, mean of two measurements to nearest 2 mmHg)*

fP-Glucose (Hemocue^®^): mmol/l Body temperature: _________^o^ C

Bioimpedance: - RZ - XC

**Venous blood sampling**:

Blood status (Hb, LPK, TPK, EVF, MPV)

fP-Glucose

B-HbA1c

Lipid status (Triglycerides, Cholesterol, LDL, HDL)

Liver status (ASAT, ALAT, ALP, Bilirubin)

Salt balance (Na, K, Ca, Creatinine)

Hepatitis B/HIV

Hs-CRP

Free T4 and TSH

**Pathophysiology associated with overweight and type 2 diabetes**

Intermediate visit

Allocation number: _________

Date: ____________________________

Investigator:________________________

Blood pressure: / mmHg (*supine left arm, after 10 min rest, mean of two measurements to nearest 2 mmHg)*

fP-Glucose (Hemocue^®^): mmol/l

**Pathophysiology associated with overweight and type 2 diabetes**

Visit 2

Allocation number: _________

Date: ____________________________

Investigator:________________________

Yes No

No medication for the last 10 days? [ ] [ ]

No ASA/NSAIDs for the last 10 days? [ ] [ ]

Fasting since 10 pm the evening before? [ ] [ ]

Followed diet recommendation? [ ] [ ]

No supplements for the last 10 days? [ ] [ ] ^E.g. omega-3 fatty acids / probiotics^

No alcohol during the last 48 hours? [ ] [ ]

No exercise during the last 48 hours? [ ] [ ]

Are you in a habitual state? [ ] [ ]

Comments: _________________________________________________________________

___________________________________________________________________________

Blood pressure: / mmHg (*supine left arm, after 10 min rest, mean of two measurements to nearest 2 mmHg)*

**Temperature:**_______^o^C

**Venous blood sampling**:

Blood status (Hb, LPK, TPK)

fp-Glucose

B-HbA1c

S-Insulin

Extra serum and EDTA-plasma for glycerol and lactate

Hs-CRP

12 ACD-tubes for thrombocyte preparation, taken with butterfly needle

**Microdialysis:**

Four thin catheters are inserted into abdominal subcutaneous adipose tissue and two thin catheters are inserted in a similar manner into an underarm muscle (m brachioradialis) to measure extracellular tissue concentrations of insulin, glycerol and lactate.

Dialysate is sampled every 15-30 min depending on placement (every 30 min in the muscle). This is performed in parallel with the blood sampling during the OGTT. An intravenous infusion with inulin (a carbohydrate which is excreted through the kidney) is started in the morning and is used as a reference for assessment of the interstitial insulin recovery in adipose tissue and muscle.

**OGTT:**

The participant drinks 75 g glucose in 2 dl water and measurements of glucose, insulin, glycerol and lactate is performed every 30 min through venous sampling during 3 hours. Participants lay in a bed during the whole day when the samplings are performed.

**Blood flow measurement in subcutaneous adipose tissue:**

A low dose of radioactive Xenon in gaseous form are injected at two spots in the subcutaneous tissue on both sites of the umbilicus, and two detectors are placed over the two depots to measure Xenon-clearance in the adipose tissue during 4 hours.

**Pathophysiology associated with overweight and type 2 diabetes**

Visit 3

Allocation number: _________

Date: ____________________________

Investigator: ________________________

Blood pressure: / mm Hg (*supine left arm, after 10 min rest, mean of two measurements to nearest 2 mmHg)*

**Weight**: ________kg **Waist**: ________cm

**Temp:** _______ ^o^ C

**EndoPAT:**__________________________________________________________________

___________________________________________________________________________

___________________________________________________________________________

Five minutes of measurement constitutes the baseline, then 5 min at 200 mmHg or alternatively 60 mmHg above the systolic blood pressure. Thereafter a 5 min measurement without stasis. This is performed to measure capillary recovery (endothelial function).

**Local anesthesia:** 10 mL Xylocain (10 mg/mL)

**Sc adipose tissue biopsy (time):** _________________________________

fP-Glucose (Hemocue®): __________ mmol/liter

1 extra 6 ml SST-tube and 2 extra 4 ml heparin tubes for biobanking.
