# Supplementary material for: Hyperinsulinemia and insulin resistance in the obese may develop as part of a homeostatic response to elevated free fatty acids: A mechanistic case-control and a population-based cohort study
Source: eBioMedicine. 2021 Mar 9;65:103264. doi: 10.1016/j.ebiom.2021.103264 (PMC7992078; doi:10.1016/j.ebiom.2021.103264)
Supplement: Supplementary file 6 [file mmc6.pdf]

February 5, 2021

To whom it may concern

I give permission to be mentioned in writing for statistical expert advice in the paper "Hyperinsulinemia and insulin resistance in the obese may develop as part of a homeostatic response to elevated free fatty acids - A mechanistic case control and a population based cohort study by Fryk E et al. submitted to eBioMedicine for publication.

Sincerely,

Nils-G. Pehrsson

Nils-Gunnar Pehrsson
CEO/Senior Biostatistician
Statistiska Konsultgruppen
Thorild Wulffsgatan 1
SE-413 19 Göteborg
